# The Social Bifurcation of Reality: Symmetrical Construction of Knowledge in Science-Trusting and Science-Distrusting Discourses

**DOI:** 10.3389/fsoc.2022.782851

**Published:** 2022-02-09

**Authors:** Cosima Rughiniş, Michael G. Flaherty

**Affiliations:** ^1^ Faculty of Sociology and Social Work, University of Bucharest, Bucharest, Romania; ^2^ Department of Sociology, Eckerd College, St. Petersburg, FL, United States

**Keywords:** science denial, social polarization, deficit model, science work, number work, time work, emotion work, boundary work

## Abstract

This article proposes a conceptual framework to study the social bifurcation of reality in polarized science-trusting and science-distrusting lay worldviews, by analyzing and integrating five concepts: science work, number work, emotion work, time work, and boundary work. Despite the epistemological asymmetry between accounts relying on mainstream science and science-distrusting or denialist ones, there are symmetrical social processes contributing to the construction of lay discourses. Through conceptual analysis, we synthesize an alternative to the deficit model of contrarian discourses, replacing the model of social actors as “defective scientists” with a focus on their culturally competent agency. The proposed framework is useful for observing the parallel construction of polarized realities in interaction and their ongoing articulation through hinge objects, such as vaccines, seatbelts, guns, or sanitary masks in the Covid-19 context. We illustrate the framework through a comparative approach, presenting arguments and memes from contemporary online media in two controversies: namely, vaccine-trusting versus vaccine-distrusting views and Covid-convinced versus Covid-suspicious discourses.

## Introduction

**FIGURE 1:**
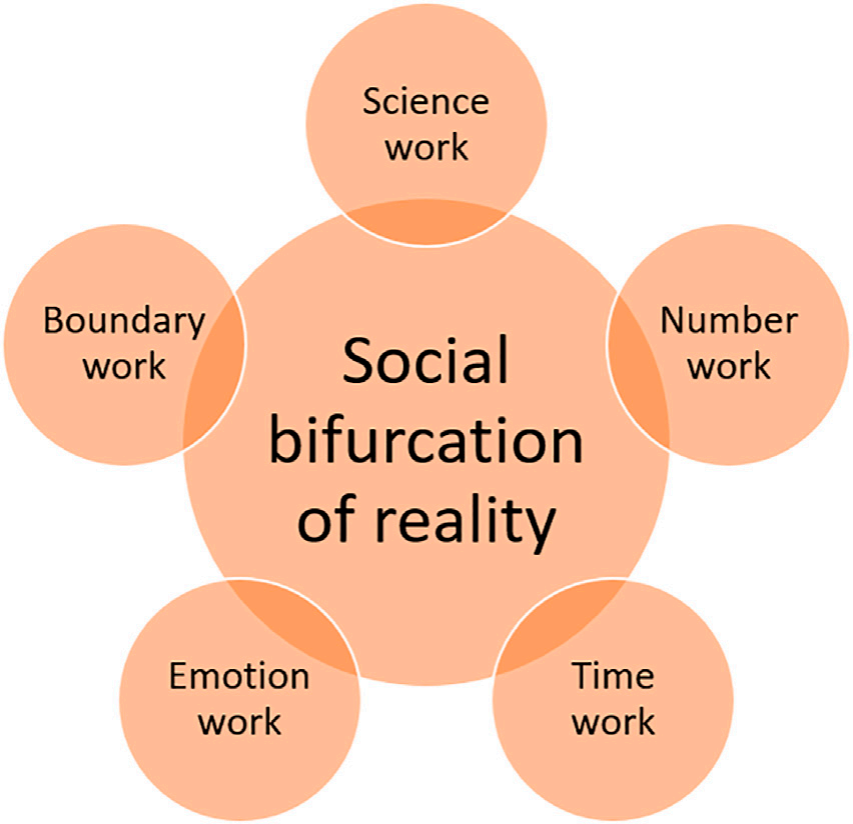
Five types of social agency symmetrically involved in science-distrusting and science-trusting social construction of knowledge.

Controversies on the merits and risks of vaccination, and the reality, causes, and proper tackling of climate change or pandemics, have become global matters of contention with potentially devastating effects. Through their reach and risks for the survival of human civilization as we know it, these controversies may soon displace past wars. While mainstream scientific evidence weighs heavily in these debates, contrarian and denialist discourses have achieved great popularity, evolving rapidly and diffusing at scale. In this article we critique previous efforts at understanding the construction of lay science-distrusting and science-trusting discourses in the so-called “deficit model” (Gross, 1994; Miller, 2001), and we propose and illustrate a conceptual template for explaining the symmetrical generation of such bifurcated realities (see Figure 1). Our examples will focus on vaccination and Covid-19, yet the template can be fruitfully applied to many past and present controversies involving scientific evidence, including seatbelts, tobacco products, AIDS, gun control, cannabis, homeopathy, or the Flat Earth movement.

Personal outlooks may be science-trusting on some issues and science-distrusting or denialist on others, at the same time. Individual views on any contentious issue may be more or less complex, coherent, or certain. They could be positioned on a broad continuum from *science denial*, typical of the more visible communities such as flat-earthers, the militant anti-vaxx, or climate-change denying activists, to a grey zone of *selective distrust of mainstream science and engagement* with scientific controversies, which merges and continues with variable forms and degrees of *trust in science and the scientific consensus*. Beyond individual configurations of belief, we can identify the ideal types (Weber, 1978) of two public configurations of meaning: namely, the poles of a science-trusting zone, at one end, and a contrarian and denialist zone, at the other, for any given issue. In what follows we highlight how these contrasting repertoires of meaning that underlie individual beliefs and emotions are constructed through five forms of social interaction: science work, number work, time work, emotion work, and boundary work.

We opt for the study of the binary, contrastive ideal types of the science-trusting and the contrarian/denialist lay worldviews, rather than the nuanced variety of their empirical combinations in individual beliefs, following Weber’s (1978) argument that ideal types, though distinct from any empirical organization of meaning, are needed to understand social reality: “Theoretical differentiation is possible in sociology only in terms of ideal or pure types” (p. 20). As he argues, subsequently, “The theoretical concepts of sociology are ideal types not only from the objective point of view, but also in their application to subjective processes. In the great majority of cases actual action goes on in a state of inarticulate half-consciousness or actual unconsciousness of its subjective meaning. The actor is more likely to ‘be aware’ of it in a vague sense than he is to ‘know’ what he is doing or be explicitly self-conscious about it. In most cases his action is governed by impulse and habit. Only occasionally and, in the uniform action of large numbers, often only in the case of a few individuals, is the subjective meaning of the action, whether rational or irrational, brought clearly into consciousness” (pp. 21–22). In accord with Weber’s perspective on ideal types, we focus on the social construction of two divergent definitions of the situation. Moreover, despite their substantive differences, we find that they are constructed by parallel processes that lead to the bifurcation of reality in public discourse and world views.

Definitions of the situation (Thomas and Thomas, 1928; Goffman, 1986; Bakker, 2016) are used, refined, and promoted by social actors of variable creativity, size, and power. They are disseminated and fine-tuned through direct and mediated interactions, through multiple media. Publics assemble around evolving symbols, coordinate through social networks of influence, and act at micro and macro levels, by selectively invoking the past, projecting the future, and assessing the present situation (Emirbayer and Sheller, 1999). With the advent of the Internet, there is an increasingly vibrant and diverse market for both science-trusting and science-distrusting symbolic constructions. The successful diffusion of the latter on digital social networks has received considerable scholarly attention (Wardle and Derakhshan, 2017; Wang et al., 2019; Johnson et al., 2020).

Denialist, contrarian, and science-suspicious discourses may be associated with socio-demographic characteristics, but they are not restricted to any social type, circulating across educational, racial, ethnic, gender, class, and generational categories (Larson et al., 2016; Hornsey et al., 2018; Bricker and Justice, 2019). As a result, in the last decades the vaccination-hesitant public has increased in size, as demonstrated by multiple studies in diverse societies (Larson et al., 2016; Hadjipanayis et al., 2020; Kennedy et al., 2011; Zingg and Siegrist, 2012; Bocquier et al., 2018; Napolitano et al., 2018). The large-scale controversy on mask wearing as a preventive measure against Covid-19 transmission is also the result of a diverse, mobilized public, who crafted a persuasive science-contrarian discourse concerning the pandemic.

In what follows we will refer to science-denialist, contrarian, and suspicious discourses on major topics with the simpler label of *science-distrusting*, following Slater et al. in acknowledging their shared apprehension of the scientific consensus and the findings of mainstream science (Slater et al., 2020). Nuances are important, and, for example, Torcello (2016) proposes to differentiate contrarian skepticism, which he calls “pseudoskepticism,” from the institutionalized skepticism of normal science. We have chosen “distrust” rather than “skepticism” in order to attend to this difference. The polarity of the ideal types of science-distrusting and science-trusting views captures well, for our purposes, the range of positions on a given issue.

The dominant conceptual frames for understanding science-distrusting discourses, including fake news, disinformation, and misinformation, rely on psychological theories of cognition, social network modelling, and political studies of propaganda (Wardle and Derakhshan, 2017; Wang et al., 2019; Mukhtar, 2020). These approaches continue to operate with an underlying *deficit model* (Gross, 1994; Miller, 2001) of public knowledge. In this model, people who take part in science-distrusting discourses are portrayed as afflicted by human cognitive limitations and emotional vulnerabilities, at turns manipulating others through their public involvement, and themselves manipulated by savvy propagandists who operate the influence machinery of digital social media. Krishna’s conceptualization of the “lacuna individual,” as a person who is “very high in his/her problem-specific motivation and activeness levels about an issue, but displays a knowledge deficiency about that issue, and high levels of negative attitudes about it” (Krishna, 2017), is a good illustration of current versions of the deficit model.

We discuss an alternative conceptual framework to the deficit model, and we explicate both science-distrusting and science-trusting public knowledge symmetrically by highlighting the individual’s epistemic agency in relation to social sources of message credibility. Social actors across the science-(dis)trust spectrum in controversies related to Covid-19, vaccination, or climate change pursue similar legitimation strategies: they seek for true facts while debunking errors and lies, they quantify yet challenge suspicious statistics, they learn from history and anticipate the future, they appeal to emotions to signal relevance and to maintain group solidarity. Starting from Simmel’s distinction between societal form and content (Simmel, 1909), we can observe parallel forms of interaction that generate and legitimize bifurcated realities with contrastive social contents. As he notes, “similar forms of socialization occur with quite dissimilar content, for wholly dissimilar purposes. (…) In the case of human associations which are the most unlike imaginable in purposes and in total meaning, we find nevertheless similar formal relationships between the individuals” (p. 299).

*Lay actors*’ *symmetrical search for information in their social circles does not guarantee symmetrical epistemic success*. We do not claim that science-distrusting and science-trusting discourses are equally valid in their claims. There are significant epistemological differences between the two discourses and their respective ways of invoking, weighting, and synthesizing evidence, as we discuss below in the section on science work. While the science-trusting discourse relies on mainstream scientific hierarchies of evidence and a strong emphasis on the distinction between causality and correlation or coincidence, the science-distrusting discourse operates with an epistemology of suspicion towards scientific authority, which privileges the truth of all plausible individual accounts, expert opinions, and studies that support their dissenting views. As scientists ourselves, we extend the benefit of trust to the scientific hierarchy of evidence and synthesized consensus, as a rule. Still, we argue, in line with scholars from sociology of knowledge (Berger and Thomas, 1966; Miller, 2001) and from rhetorical studies (Gross, 1994), that a better appreciation of the symmetrical processes of lay knowledge-building across divergent discourses will better incentivize the scientific community to engage the general public more effectively. Our critique of the deficit model highlights the central role of *trust* in accepting or rejecting the findings of mainstream science. An appreciation of the symmetrical processes of world-building in operation across the spectrum of trust can also redefine what counts as *marked vs. unmarked views*, or *normal vs. abnormal views* (Zerubavel, 2018), especially in the scientific community. Sustained partnerships and horizontal dialogues, rather than paternalistic attempts to enlighten the public as regards scientific literacy, stand a better chance of fostering confidence in mainstream science and expert organizations. For both science-distrusting and science-trusting worldviews, the crux of the matter is whether to trust a specific, here-and-now, historically contingent social organization of science (not an abstract scientific methodology) in its ability to withstand the pressure of human bias and organized commercial and political interests.

Our primary contribution in this article consists in analyzing five sensitizing concepts and assembling a conceptual template for *explicating the symmetrical social construction of public bifurcated knowledge*. We discuss, integrate, and illustrate notions that formalize lay social actors’ acts of knowledge-making: science-work or staging science (Degele, 2005), number work or quantification (Espeland and Stevens, 2009), time work (Flaherty, 2003; Flaherty, 2011), emotion work (Hochschild, 1979), and boundary work or maintenance (Lamont and Molnár, 2002), or what Erikson calls “social speciation” (Erikson, 2017). These five interrelated concepts can be understood as manifestations of *social skills*, or the “abilities to engage others in collective action (…) that proves pivotal to the construction or reproduction of local social orders” (Fligstein, 2001, p. 106). These types of knowledge-making acts constitute shared orders of evidence, temporality, emotionality, and community membership that enable further coordinated action.

Secondly, we thus offer an alternative to *the deficit model in studying science-distrusting beliefs*. Through the proposed template, we highlight and facilitate the symmetrical study of science-compatible and contrarian worldviews, following the symmetry principle advanced by Bloor (1976). While most research focuses on explaining the marked, science-contrarian discourses, we facilitate the study and understanding of what is, in the academic community, the unmarked (Brekhus, 1998; Zerubavel, 2018), namely science-trusting discourses. Specifically, we facilitate the observation and conceptualization of how contrarian discourses also invoke science, expertise, and numerical evidence in the pursuit of facts, and, conversely, how science-trusting public discourses also employ emotional appeals and public shaming, among other tactics of worldview consolidation.

This conceptual template challenges a blanket invocation of postmodernism to explain such controversies (Brown, 1990; Smith, 1996; Flaherty et al., 2002; Kata, 2012; Bricker and Justice, 2019). We argue that science-contrarian and science-trustful public discourses often share a commitment to finding a unique, factual, and certain truth and debunking false narratives through critical thinking and investigation, in stark contrast to epistemic relativism specific of postmodern epistemologies. While postmodernism is relevant for understanding the social forces that eroded the hegemonic status of science in present-day societies, it is important to keep in mind that members of contrarian or science-denialist groups are not necessarily identifying or identifiable with a postmodern, relativistic attitude (Hausman, 2019). Indeed, they typically reject cultural relativism.

## Overcoming the Deficit Model of Explaining Science-Distrust

Social order depends on shared cognitive resources or knowledge (Ramírez-i-Ollé, 2018). Yet, scientific evidence does not directly determine popular beliefs. Scientific ideas and findings have often been contested and controversial throughout history. In recent decades we can observe large scale manufacture of doubt by organized interests (Orekes and Conway, 2010; Goldberg and Vandenberg, 2019; Jamison et al., 2020; Michaels, 2020) and reconfigurations of expertise in an increasingly individualizing reflexive modernity (Prior, 2003; Collins and Evans, 2008; Eyal, 2013; Scott, 2016; Carrion, 2018; Yoo, 2018). These processes reconfigure social actors’ relations to science, expertise, and other sources of representations of reality, leading to the emergence of alternative and polarized forms of public knowledge on matters of public interest.

A systematic and persistent difficulty in studies of public engagement with science and scientific controversies consists in overcoming the so-called *deficit model*, which portrays the public as “faulty scientists” (Wynne, 1996; Locke, 2002; Michael, 2002). The deficit model explains science-contrarian and denialist beliefs through cognitive shortcomings or vulnerability to manipulation, in opposition to science-trusting beliefs which are framed as cognitively normal and self-explanatory. The principle of explaining difference through difference encourages scientists to focus on the marked contrarian beliefs, and to search for cognitive particularities of those who hold them, while the academically unmarked science-compatible beliefs and their holders remain a taken-for-granted benchmark. To illustrate, a systematic review of research on why people believe COVID misinformation highlights the role of cognitive limitations that predispose people to reject expert information, and the influence of political manipulation (Mukhtar, 2020), while a comparative survey on beliefs in COVID misinformation identifies its correlation with low numeracy and trust in scientists (Roozenbeek et al., 2020). The social actions of defining the situation, involved in mobilizing publics around credible arguments, be they compatible or incompatible with the scientific consensus, remain less examined.

Science and social order are co-constituted, with significantly more heterogeneity and interdependence than in a dichotomous deficit model (Michael, 2002). The scientific method and the institution of science have systematic and distinctive properties of adjusting beliefs to their external objects and converting them in effective technologies. Still, social systems have proven time and again to be very flexible in incorporating feedback-resistant beliefs in their functioning. This indicates that considering legitimation processes at social level, in addition to cognitive biases at individual level, will better explain social-wide adoption of science-distrusting beliefs. Yet, the deficit model is persistent both in scientific literature and in public discourses, due to its advantages in formulating research programs and public policies (Simis et al., 2016). Both the attractiveness of the deficit model and the call to move beyond it in research projects and in science teaching and communication are widely acknowledged. We find them in studies of vaccination skepticism (Blume, 2006; Hobson-West, 2003; Goldenberg, 2016; Lawrence, 2016; Kitta and Goldberg, 2017; Bricker, and Justice, 2019; Vulpe. 2020; Vulpe, 2021) and in studies of public beliefs and attitudes towards climate change (Bulkeley, 2000; Stoutenborough and Arnold, 2014; Suldovsky, 2017; Plutzer and Hannah, 2018).

As regards anti-vaccination, studies anchored in *a deficit model* have documented individual personality traits and cognitive properties that raise the risk of espousing science-contrarian beliefs and attitudes. Vaccine-distrusting people may on average display different cognitive styles (Poland and Poland, 2011) and ideological commitments (Hornsey et al., 2018) than people who trust vaccination. Vaccine distrust is statistically associated with biased risk processing (LaCour and Davis, 2020) and a specific psychological pattern comprising higher conspiratorial thinking, reactance, disgust towards blood and needles, and individualistic/hierarchical worldviews (Hornsey et al., 2018). Vaccine-distrusting parents are characterized through an unwillingness to engage with scientific evidence (Browne et al., 2015), and are described as guided by emotions and intuitive thinking, in contradistinction to analytically rational thinking (Tomljenovic et al., 2020). Nevertheless, the social normalization of vaccine-distrusting and other contrarian beliefs, especially their reach and penetration in all social strata, cannot be entirely accounted for by individual cognitive or psychological features. The classic critiques of trait theory in sociology of deviance are also relevant in this respect. As Becker observed, many who exhibit the deviant behavior in question do not have the trait, and many who do have the trait do not exhibit the deviance in question; moreover, there is great variability across time among those who engage in the studied behavior (Becker, 1963). We need to examine the *broader cultural dispositions, changing social structures, and actions* of consolidation and innovation in the market for science-distrusting ideas.

As regards *cultural dispositions and social structures*, studies point to historical changes accounting for the rise of science distrust through new sources of legitimacy, from neoliberal capitalism (Reich, 2014; Sanders and Burnett, 2019), a rise in relevance of conspiracy thinking as a defense of individualism (Melley, 2002), and the creation of the “informed patient” (Kata, 2010) asking for individualized treatment (Ciocănel, 2016), to the evolution of the online environment in which anti-vaccine accounts are densely interlinked (Kata, 2012; Numerato et al., 2019; Johnson et al., 2020). The internet and, particularly, the Web 2.0 have boosted the diversity, visibility, and circulation of vaccine hesitancy and of science-contrarian messages, in general.

In this paper we adopt an *agentic* perspective in answering the call for overcoming the deficit model in studies of science-distrusting beliefs (Bulkeley, 2000; Hobson-West, 2003; Blume, 2006; Stoutenborough and Arnold, 2014; Goldenberg, 2016; Lawrence, 2016; Kitta and Goldberg, 2017; Suldovsky, 2017; Plutzer and Hannah, 2018; Bricker and Justice, 2019). We start from Emirbayer and Mische’s conceptualization of agency as “the temporally constructed engagement by actors of different structural environments (…) which, through the interplay of habit, imagination, and judgment, both reproduces and transforms those structures in interactive response to the problems posed by changing historical situations” (Emirbayer and Mische, 1998, p. 970). Thus, we contribute to the study of the symmetrical construction of science-distrusting and trusting discourses, through *social actions that mobilize typical sources of legitimacy, project and reject possible futures, and continuously evaluate ongoing events, defining situations and reacting to them*.

An agentic perspective is especially useful for observing the large scale, collective, distributed knowledge-making work on social media and collaborative digital platforms. Participants in digital forums, groups, and threads contribute through acts of knowledge-making and sharing to the social construction of reality, mediated by algorithmic architectures. Therefore, this conceptual template facilitates the observation, classification, and theorizing of collective knowledge making, both in face-to-face and mediated interactions.

## A Conceptual Template That Accounts for the Social Bifurcation of Reality

In what follows we analyze and we synthesize *five sensitizing concepts* relevant for the study of symmetrical knowledge-making processes leading to bifurcated realities. Our toolkit includes concepts that capture an important agentic dimension of the social construction of knowledge: science work or staging science, number work or quantification, time work, emotion work, and boundary work or maintenance. We start with a brief discussion of the template and an illustration regarding vaccination, and we go on to a discussion on how the template captures the Covid-suspicious/Covid-convinced polarity.

Our template is particularly useful for unpacking the *reason/emotion* divide that is characteristic of the deficit model. Science-distrusting discourses are often described through their appeals to emotions and community building, while science-trusting discourses are portrayed as factual, thus rational. Still, a constructivist perspective on public knowledge enables us to observe parallel (though not identical) work of fact-making and emotion and identity work in both worldviews (Fischer, 2019; Fischer, 2020; Durnová, 2018, Durnová, 2019).

Firstly, there is symmetrical effort at *science work* or *staging science* (Degele, 2005) by invoking its authority through different tactics. Given the strong legitimacy of science in the modern world, communities who seek to advance their views in a competition with others stand to benefit from appealing to scientific evidence or expert opinion, claiming scientific validation at least for some elements of their definition of the situation. Creators and consumers of science-contrarian or science-denying content symmetrically invoke the values of critical thinking, independent research, and evidence-based action, even when operating with lay epistemologies different from those currently in scientific use (Prior, 2003; Hobson-West and Hons, 2005; Doty, 2015; Attwell et al., 2018; Carrion, 2018). Tactics for science work are adapted to the situation. While science-trusting discourses preferentially invoke the *scientific consensus* and mainstream views, science-distrusting discourses invoke *contrarian scientists*, who are positioned as heroes fighting against a repressive regime of truth. While both types of discourse symmetrically invoke scientific evidence, what counts as evidence and, especially, what matters in weighting and synthesizing conflicting pieces of evidence is different. Mainstream science operates with models of levels of evidence, or *evidence pyramids* (Mulimani, 2017), that separate anecdotal experiences and opinions from research and classifies science according to methods’ strength and volume of data. In the mainstream scientific approach, individual experiences count as data points, but not as evidence per se, and scientific findings are weighed differentially, according to criteria that differentiate the value of evidence. Alternatively, contrarian discourses employ *a flat structure of evidence*. In this approach, individual experiences count as direct evidence, no matter whether they are validated, ignored, or invalidated by scientific or political authorities. Primary, empirical studies that support the contrarian position are invoked as definitive scientific facts, even more so when they are downplayed in the scientific work of weighting and aggregation that creates the scientific mainstream. Contrarian discourses disregard the systematic analyses and syntheses that generate the scientific consensus, in favor of a curated collection of personal and expert testimonies and individual studies, which are often situated at the lower levels of evidence in the organization of mainstream science, or even retracted.

Secondly, there is symmetrical *number work*. *Quantification,* “the production and communication of numbers” (Espeland and Stevens, 2009, p. 402) is “a constitutive feature of modern science and social organization” (*idem*). Numbers, statistics, and mathematical models are shaping our lives at many layers (Matei and Preda, 2019). Still, while contemporary societies put great confidence in numbers, there is also systematic skepticism and wariness of “how to lie with statistics” (Steele, 2005). Numbers are simultaneously trusted and mistrusted; they are used, resisted, and abused. We can observe frequent appeals to numbers, in both science-skeptical and science-trusting discourses, only there are different numbers and different tactics of use, too (Billig, 2021). What is a good-enough quantification for some, may be presented as a complete error, fraud, or manipulation for others. While everybody accepts that numbers are approximations, some choose to go along with them, while others choose to reinterpret, reject, or maybe replace them. Science-trusting discourses benefit from the large infrastructure of quantification provided by the scientific enterprise. Science-distrusting ones may have their own, alternative resources for generating numbers, they may select and reinterpret numbers from the scientific infrastructures, or they may reject numbers by appealing to individual testimonies.

Thirdly, sensemaking in both science-distrusting and science-trusting discourses relies on the construction of histories and the projection of futures, on the cumulative addition of events, on significant sequences that prove or disprove causality, good intentions, and competence. This points to the crucial role of argumentative use or manipulation of time in advancing and defending knowledge claims. In its original formulation, the concept of *time work,* understood as “one’s effort to promote or suppress a particular temporal experience” (Flaherty, 2003, p. 19), sheds light on how people attempt to control or customize various dimensions of time in diverse social contexts (Flaherty, 2011; Dalsgård et al., 2014; Flaherty et al., 2020), from managing health care (McCoy, 2009) to homeschooling (Lois, 2010) or to maintaining long distance relationships (Jurkane-Hobein, 2015). The concept of *argumentative time work* (Ciocănel et al., 2020; Rughiniş et al., 2020) reveals legitimation tactics that make use of shared temporal expectations and valorizations to create plausible representations of reality.

Fourthly, *emotion work* or “the act of trying to change in degree or quality an emotion or feeling” (Hochschild, 1979, p. 561), is part and parcel of knowledge work. As James noted in his 1890 work on “The Principles of Psychology,” “In its inner nature, belief, or the sense of reality, is a sort of feeling more allied with the emotions than to anything else” (James, 1982, p. 158). Facts stir and justify emotions, and emotions establish the relevance of facts, create meaning, and orient action in the fast thinking of daily life (Massey, 2002; Jasper, 2014). Emotion is constitutive for reason (Damasio, 2005), and feeling rules are constitutive to the definition of the situation and the social institutions that regulate it. Communication of science requires emotion work (Davies, 2019). Both science-distrusting and science-trusting discourses shape specific *feeling rules* and *emotion accounts*, socializing members to express appropriate emotions and to resist the wrong emotional urges. There is symmetrical yet different allocation of fear, hope, anger, indignation, and admiration, among others, as we will exemplify below. Through repeated acts of inciting and discouraging emotions, actors in science-distrusting and trusting publics contribute to emotional amplification through feedback (Hallett, 2003) and to *emotional socialization* (Thoits, 2004) through the constitution and change of feeling rules (Hochschild, 1979) and accounts.

Finally, perhaps the most visible form of symmetry between the science-distrusting and science-trusting publics consists in *boundary work or maintenance* (Lamont and Molnár, 2002; Pachucki et al., 2007; Erikson, 2017). For both, the out-group elite is denounced as manipulative and incompetent, the out-group scientists are denounced as corrupt or biased, and the out-group members are denounced as stupid, uncritical, and brainwashed, captive under the spell of powerful indoctrinating narratives, as we illustrate in the next sections dedicated to the vaccination and Covid-19 controversies.

The bifurcated social realities that emerge through these parallel forms of social interaction may be articulated through specific *hinge objects* that have common, circulating aspects across worldviews, while also acquiring polarized, contrastive interpretations and versions–such as cigarettes, vaccines, or sanitary masks. These act as the mirror in Lewis Carrol’s “Through the Looking Glass,” which reverses the logic of life, while keeping the two worlds in close interaction and mutual interdependence. Hinge objects combine shared elements, including material and symbolic parts, with polarized interpretations. The cigarette may be a toxic tool of Big Tobacco’s commercial exploitation in one worldview, and a business-as-usual way of relieving stress in the other. Vaccines may be construed as life-saving inventions of medical science and technology, or, alternatively, as dangerous substances that degrade the immune system and consolidate people’s dependence on Big Pharma. Masks can be viewed as simple instruments for protecting oneself and others from deadly pathogens with the minor cost of discomfort, or as toxic, suffocating symbols of manipulation and disempowerment that global elites use to consolidate their power. These objects include shared material and symbolic elements that can be produced, modified, circulated, and used across worldviews, but they are also a focus of intense and heavily divergent work of sense-making through science work, number work, time work, emotion work, and boundary work.

*Hinge objects* can be conceptualized in contradistinction to the concept of “boundary objects” (Star and Griesemer, 1989; Star, 2010). Boundary objects articulate different communities of practice, enabling them to collaborate in the absence of consensus. In contrast, hinge objects connect conflicting communities of practice, enabling them to better specify their worldview in opposition to one another. These controversial objects are gradually created through processes of *formation* (Hirschman and Reed, 2014), marked by contingency as well as the agency and alliances of various social actors.

In the next sections we illustrate the social bifurcation of reality in the case of the vaccination and the Covid-19 controversies, and *we use memes and other social media messages to illustrate the binary ideal-typical worldviews that define these tensions*. We have selected memes and posts simply for didactic purposes and for their potential of capturing persuasive arguments (Flaherty and Rughiniş, 2021), including a small selection of items that best instantiate the forms of science work, number work, time work, emotion work, and boundary work, without attempting to capture the vast diversity of memes that circulate around these topics.

After the debut of the pandemic in December 2019, we have witnessed an increasingly polarized collective sensemaking regarding the causes, effects, and ways to approach this disorder. There has been considerable uncertainty in all professional communities as to what is happening, and what is to be done. Scientific research has grappled with the challenge of describing the key parameters of the novel coronavirus and its social impact. Gradually, two definitions of the epidemiological situation have emerged and evolved in mutual differentiation. These two definitions of the Covid-19 disorder have acquired relatively stable contours. They are usually identified through the contrast between a scientific or expert worldview, and a negational one. We have chosen to refer to them as the *corona-convinced* and the *corona-suspicious* definitions, highlighting their contrastive explanations of the roots of the disorder.

To sum up the general outlines, in the corona-convinced definition of the situation we are facing a pandemic caused by the novel coronavirus SARS-Cov-2. The virus is highly contagious, with a case fatality significantly higher than the seasonal flu, estimated at between 1% and 5% in various countries in December 2020 and at about 2% globally in December 2021 (Ritchie et al., 2020). It originates in the Wuhan region of China, possibly transmitted from bats, or created in a lab. Face masks, both surgical and textile varieties, are an effective tool to prevent transmission. People who do not acknowledge these facts are dubbed “covidiots.” Conversely, in the corona-suspicious definition, we are facing a disorder caused by globalizing elites trying to manipulate the people through an invented or exaggerated pandemic. The novel coronavirus, often referred to as the China or Wuhan virus, is not the true cause of the disorder. This is a social rather than a true epidemiological upheaval. It is thus a “plandemic,” to use the term launched by the viral video with the same title in May 2020 (Nazar and Pieters, 2021), weaponized by political interests through mainstream media (MSM) manipulation. Telling anticipations of this pandemic can be read in Bill Gates’ 2015 TED discourse, or in Dean Koontz’ 1981 novel. The virus is similar to the seasonal flu, or even less contagious and/or fatal. It is originating from China, but it may also be fictive, or created in a lab. Face masks are seen as inconvenient and useless, or downright dangerous. Mask requirements infringe on individual freedom. They are a means of instilling obedience in people who wear them, dubbed as “sheeple,” or even of increasing the rate of infections by damaging wearers’ immune systems, in order to promote vaccination, to enhance global surveillance policies, and to enable a “Great Reset” of capitalism.

There is a correlated, yet distinctive controversy concerning the appropriate policy response to the pandemic, centered on estimating the cost/benefit ratio of lockdowns and various restrictions on mobility and economic activity. The Great Barrington Declaration (Kulldorff et al., 2020) and the John Snow Memorandum (Alwan et al., 2020) synthesize this policy divergence. While the Great Barrington Declaration is also endorsed by people who dispute the severity or the reality of the pandemic, the Declaration itself does not make or rely on statements about the causes and gravity of Covid-19, addressing instead the morally appropriate social reaction. Thus, we did not include this policy divergence in our discussion of alternative Covid realities, as our focus consists in bifurcated ontologies.

## Science Work

Both sides of the vaccination controversy symmetrically and rhetorically (yet differently) *work with science* insofar as they invoke experts who support, with variable degrees of legitimacy, honesty, quality, and intensity, their opposing points of view. For example, a study of the persuasion tactics involved in online anti-vaccination sites found widespread use of “expert opinion,” including opinions of people with expert or scientific titles, or references to studies published in scientific journals, or at least journals appearing to be scientific: of the 480 sites included in the study, use of expert opinion was found in almost 60% of them (Moran et al., 2016). Andrew Wakefield, the author of the infamously retracted study claiming a connection between the MMR vaccine and autism, is cited in anti-vaccination discourses as a “persecuted scientific hero” (Bricker and Justice, 2019). There are appeals to science and displays of scientific savvy (Poltorak et al., 2005) on both sides of the debate on the link between the MMR vaccine and autism, though the underlying understandings and misunderstandings of science are different (Scott, 2016). Kata’s review of the communication in vaccination-skeptical discourses identifies a “skewing the science” tactic, consisting in cherry-picked references, and the appeals to “brave maverick doctors” (Kata, 2012). To further illustrate staging science in vaccination controversies, Green discusses anti-vaxx memes’ appeal to science (Green, 2017), and Coleman presents the anti-vaxx public’s heroic epistemology of liberation from a false scientific paradigm (Coleman, 2017).

*Science is rhetorically deployed* by both the corona-convinced and the corona-suspicious publics. In the first, there are continuous references to the flow of research findings, syntheses of evidence, and discussions of the emerging scientific consensus, in relation to the EBM pyramid of evidence and other classifications of scientific authority (preprints/publications, prestigious/less prestigious journals). Science is seen as collaborative, distributed, and institutionalized. In the corona-suspicious public, there are also continuous references to compatible scientific studies and experts. Sometimes, but not always, they have a longstanding contrarian profile, or publish in fringe or predatory journals. The emerging scientific consensus is as a rule challenged, ignored, or denied. Shifts in the scientific consensus (for example concerning the preventive use of face masks) is denounced as a sign of incompetence and manipulation. There is no model of a hierarchy of evidence. There is a romantic, individualistic, heroic portrayal of science and scientists, and a high moral appreciation of contrarian experts. While the hierarchy of evidence in mainstream science allows for substantial changes in the emerging consensus as new research accumulates, the epistemology of suspicion specific to contrarian discourses favors constancy, since confirmation of the presumed repressed knowledge is the very criterion on which evidence is evaluated.

**FIGURE 2:**
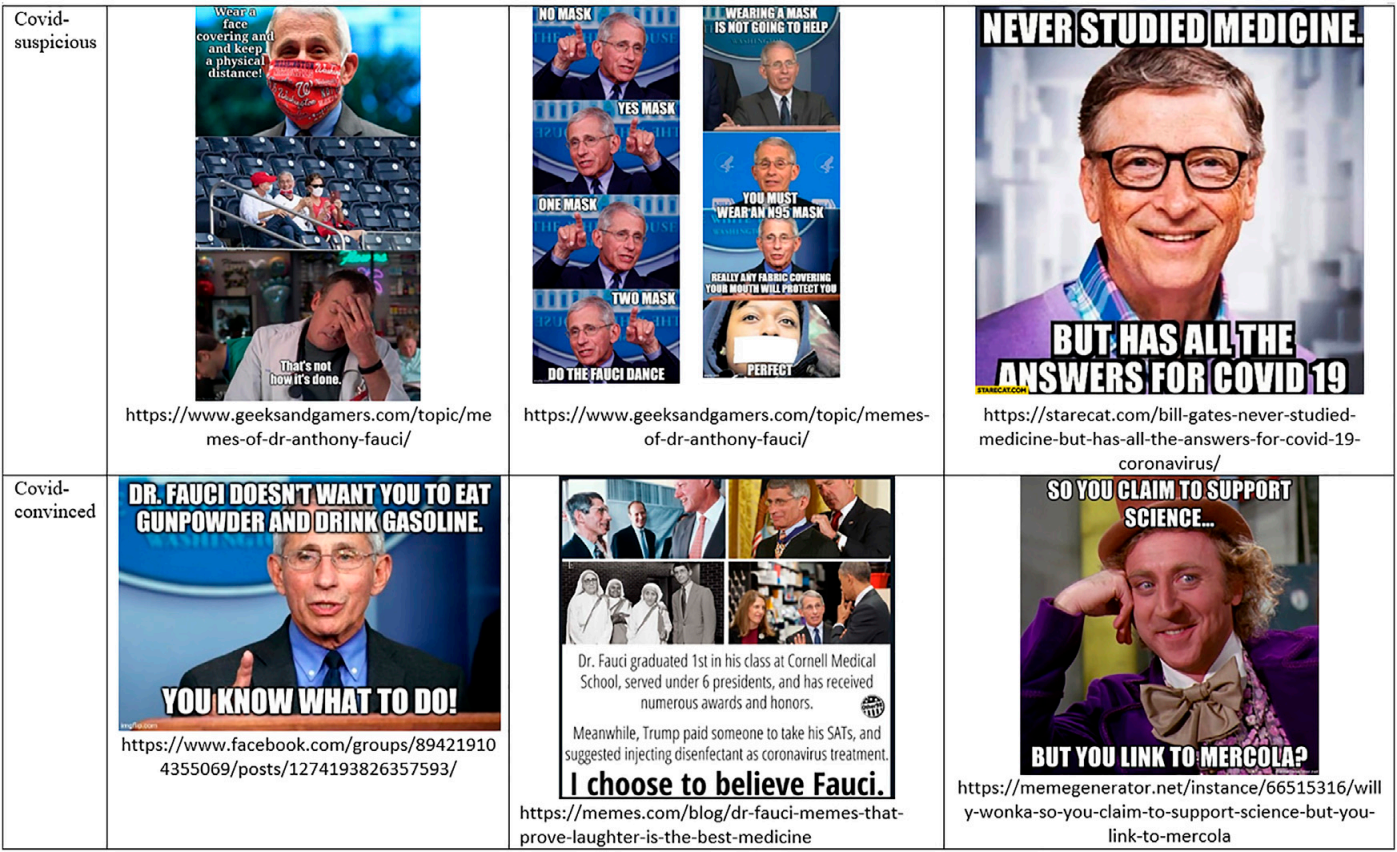
Staging science by highlighting or undermining expert voices in Covid-skeptical and Covid-anxious memes.

We notice in Figure 2 how *prominent experts become hinge objects* by articulating contrastive worldviews. Visible public figures such as Anthony Fauci and Bill Gates on the vaccine-trusting side, or Joseph Mercola and Andrew Wakefield on the vaccine-distrusting side, are either invoked as true experts or as fake, corrupt ones. The life-saving hero of one discourse is the evil perpetrator in another. Science work, emotion work, and boundary work support such polarized characterizations which, in turn, consolidate the legitimacy of the respective science-trusting or distrusting discourses.

There is a significant body of literature focusing on *the impact of postmodernism and relativism on the legitimacy of science*, particularly in social sciences (Brown, 1990; Smith, 1996; Flaherty et al., 2002). A postmodern attitude is also imputed to the knowledge-making work of antivaccine groups (Kata, 2012; Bricker and Justice, 2019). Still, anti-vaxx and vaccine-skeptical publics do not espouse a postmodern attitude, as they do not legitimize their claims through relativity or subjectivity, but through the certainty of various forms of evidence, including personal experiences and expert claims. While the flat structure of evidence in such discourses appears postmodern to external observers, members in vaccine-skeptical groups see themselves as avid pursuers of a single, objective truth, even when it describes an individual’s particular condition (Poltorak et al., 2005; Toth, 2020), rather than explorers of plural, subjective, alternative interpretations. If we take the key features of postmodern medicine to be 1) hostility towards unique truths, 2) aversion to scientific objectivity, and 3) reduced trust in expertise (Gray, 1999), then mistrust of mainstream knowledge and expertise seems to be the only common point publicly shared by anti-vaxx and other science-skeptical discourses with the postmodern attitude.

**FIGURE 3:**
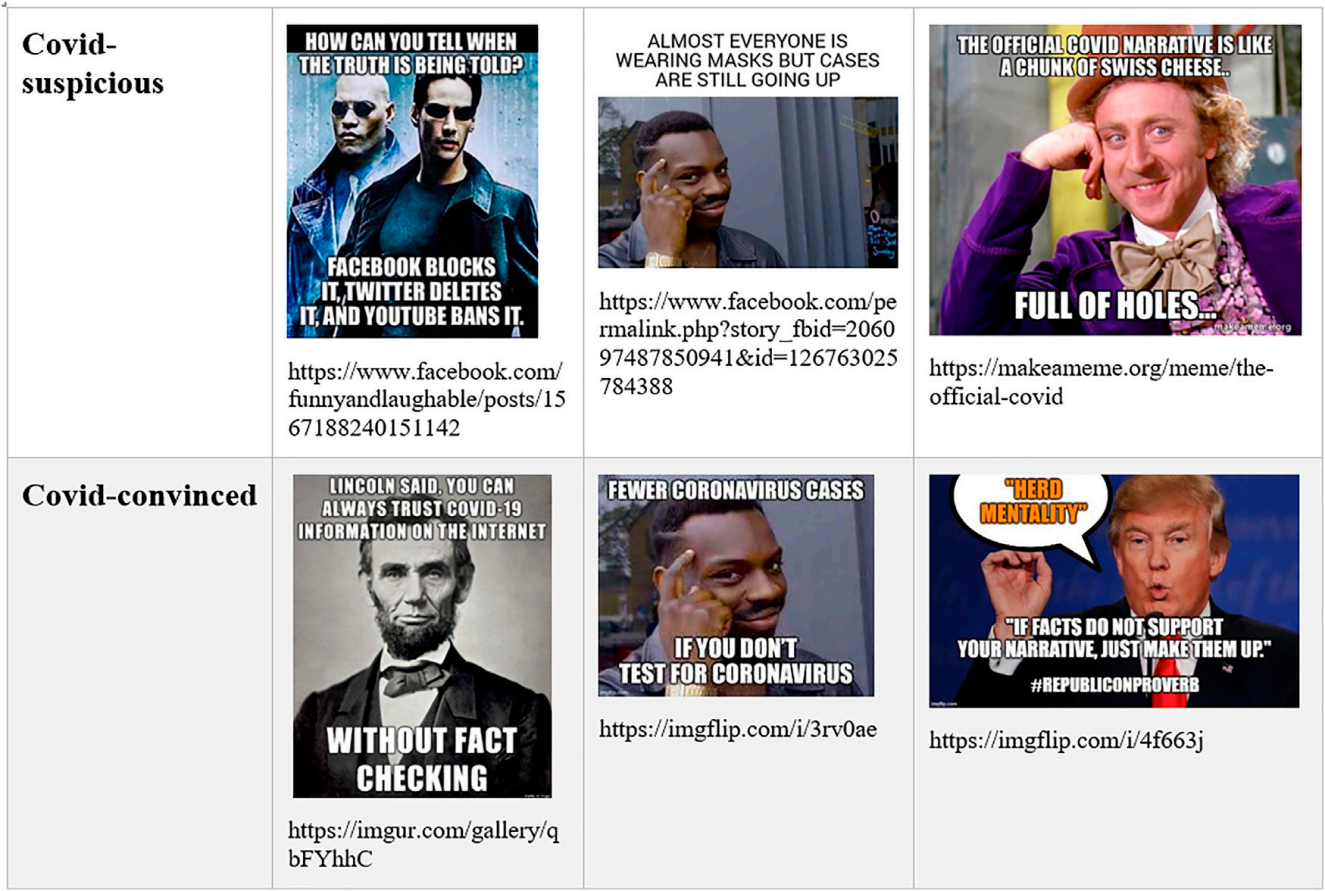
Seeking for the factual truth in Covid-suspicious and Covid-convinced memes.

Similar with vaccination debates, we see that participants in both definitions of the pandemic situation consistently claim to search for the truth, to rely on facts, and to debunk errors and manipulation. There is zero praise for epistemic relativism or for the rhetorical nature of knowledge. Both sides denounce the other side’s “narrative” as manipulative ideology, rather than a situated, legitimate version of events. The concept of “narrative” is used with negative connotations and imputed to the outgroup (see Figure 3).

## Number Work

*Numbers* that measure disease and risk are heavily traded in both vaccination-confident and vaccination-skeptical publics. While pro-vaccination arguments trust numerical estimates from mainstream scientific research, contrarian arguments often challenge them as fake and manipulative, and bring their own versions. Vaccine-distrusting and vaccine-trusting estimates of deaths related to vaccination differ by two or more orders of magnitude (CDC—Centers for Disease Control and Prevention, 2021a, Gov.uk–The Government of the United Kingdom, 2021; Smith, 2021).

**FIGURE 4:**
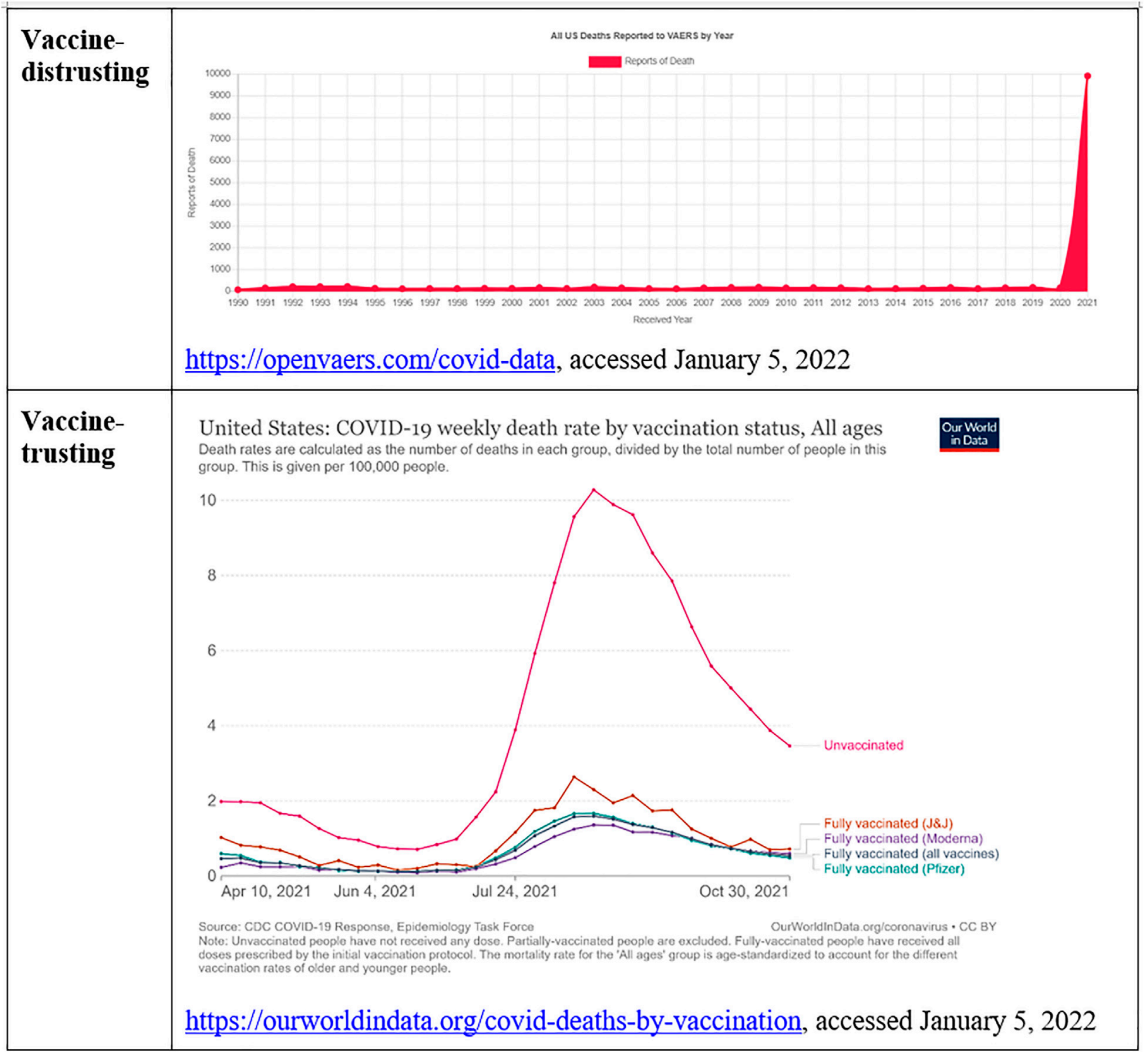
Contrastive interpretation of vaccines through number work as deadly shots (OpenVaers.com) or protection against death (OurWorldInData.org).

For example, the governmental platform VAERS—Vaccine Adverse Effect Reporting System in the UnitedStates, which offers public access to all claims of vaccine damage, has been a source for alternative estimates of vaccine risks (Coleman, 2017). Its use has increased during the Covid pandemic, in which this platform has become a powerful resource for numerical estimates of Covid-vaccine injuries in the vaccine-distrustful discourses (Motta and Stecula, 2021). The privately created platform OpenVaers.com is making such numbers easily available for vaccine-distrusting actors, and the chart illustrated in Figure 4 has received viral circulation on social media. Through and beyond such reporting platforms, there is a large-scale counting work for victims of vaccines, and web platforms publish lists of vaccine injury victims. For illustration, see the lists and numbers published by the two largest funders of antivaccination ads on Facebook (Jamison et al., 2020): both StopMandatoryVaccination.com (2020) and the antivaccine platform funded by Senator Robert F. Kennedy, Jr (Children’s Health Defense Team, 2021) present detailed estimates of vaccine-related injuries and deaths. Outside of the Covid-19 vaccine controversy, the increasing incidence of autism and auto-immune disorders plays an important role in the vaccination-distrusting arguments, which rely on studies that correlate these evolutions to an increasing prevalence of vaccination (Goes, 2013), disattending to the bulk of mainstream scientific evidence which disconfirms any causal link (Gerber and Offit, 2009).

We notice, in Figure 4, how the Covid-19 vaccine is a hinge object articulating science-distrusting and science trusting discourses through its widely divergent evaluations.

**FIGURE 5:**
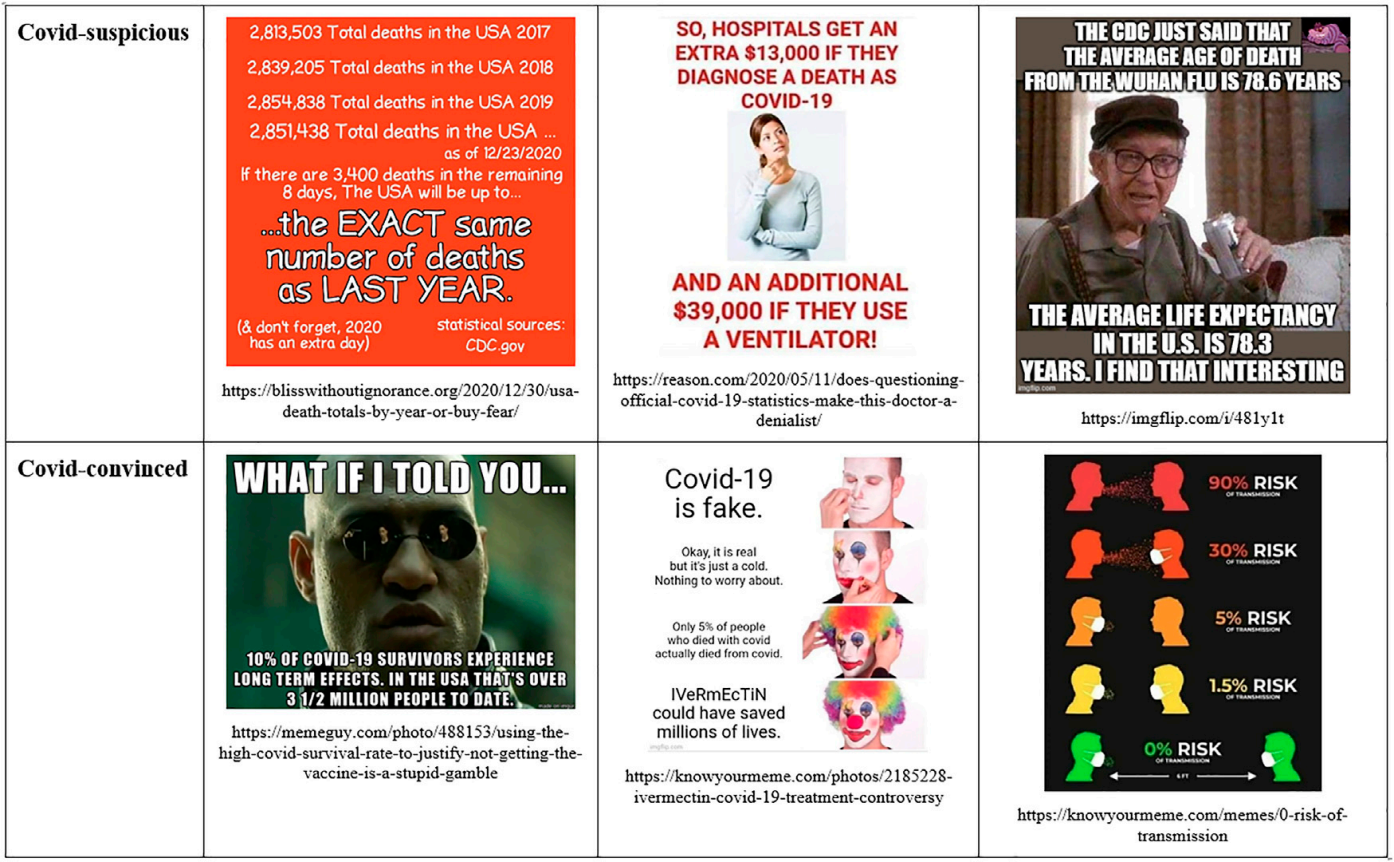
Number work in Covid-suspicious and Covid-convinced memes.

Also as regards *number work*, in the corona-anxious discourse the COVID-19 pandemic is closely monitored on indicators such as the number of new cases and total cases, number of recoveries, number of deaths, mortality and fatality rates, number of hospital admissions, and number of intensive care unit beds needed. Official and global scoreboards that track the evolution in real time are largely trusted, even if biases and other errors are expected and sanctioned. National estimates are considered comparable, despite policy differences in testing or reporting (see for example Ritchie et al., 2020). For the corona-suspicious public, official numbers and global scoreboards deserve wariness. They are taken out of context, exaggerated, manipulative, or outright false. In the Covid-suspicious discourse, Covid-19 is much less contagious and fatal than people are made to fear. Deaths from multiple sources are maliciously attributed to the coronavirus infection. In the Covid-19 suspicious view, there is no excess mortality caused by Covid-19 (see Figure 5), in contrast with official estimates of systematic excess mortality since the beginning of the pandemic (CDC—Centers for Disease Control and Prevention, 2021b). These alternatively-advanced numbers portray a quite different, more optimistic situation.

**FIGURE 6:**
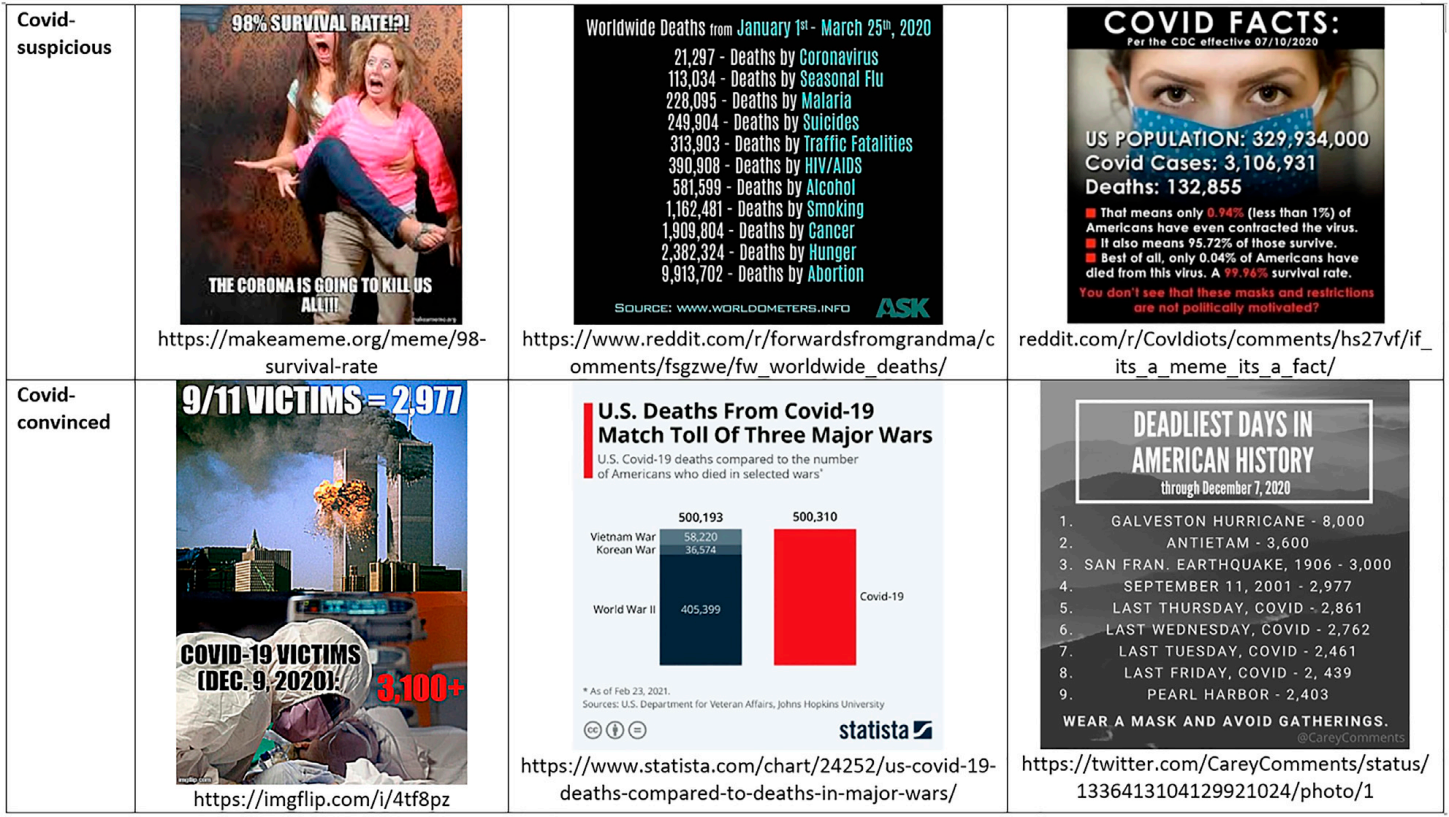
Covid death toll as hinge object connecting Covid-convinced and Covid-suspicious discourses.

Even when numbers are the same, their interpretation may be divergent. The death toll of Covid-19 functions as a symbolic hinge object, in which the same official figures acquire opposing interpretations and sustain divergent worldviews. For example, in the US Covid-convinced discourses, the Covid death toll has surpassed the 9/11 deaths and the Vietnam War deaths in 2020 (see Figure 6), and the Civil War deaths in November 2021 (Jones, 2021), marking the pandemic as a national tragedy. From the Covid-suspicious standpoint, in contrast, the coronavirus has a “98 percent” survival rate (see Figure 6).

## Time Work

The role of *sequence and coincidence* in anti-vaccination discourses also points to the importance of *argumentative time work* in shaping the two opposing definitions of the situation. Vaccination-trusting scientists denounce the “temporal confusions” of correlation with causality and the reliance on “just-so” stories of incidental vaccine damage that feature in anti-vaxx claims (Bearman, 2010; Gasparini et al., 2015). What is proof for one side is error for the other. Contrarian arguments highlight the sequential patterns of vaccination *followed by* serious health damage or death as conclusive proof that something is very wrong (Scott, 2016).

There is also considerable debate concerning vaccination *timing and schedules*. Vaccination-hesitant parents and doctors often believe that some vaccinations should be postponed to alleviate the burden for the immune system, and to better fit the circumstances of the child, while vaccination-trusting parents adhere to mainstream scientific vaccination schedules (Robison et al., 2012; Rendle and Leskinen, 2017; Rendle and Leskinen, 2017).

The vaccination-hesitant and the vaccination-trusting sometimes also inhabit *bifurcated histories*, one attributing increases in life expectancy to hygiene and nutrition, while blaming the iatrogenic risks and medical errors of biomedicine (Poltorak et al., 2005), and the other acknowledging and praising its life-saving impact.

Through *argumentative time work*, a bifurcated present is linked to contrasting pasts and diverging futures. The corona-convinced future is marked by the uncertainties of coronavirus-related disease. There are uncertain, possibly long-term consequences that have not been discovered, such as heart disease (Warraich, 2020), psychiatric disorders, or the Long-Covid. On the contrary, the corona-suspicious public is wary of risks of elite capture in the shadows of pandemic agitation, aiming for a globalist and anti-capitalist “Great Reset” of social organization. The convinced view is energized by hopes in vaccination success for creating herd immunity with lowered death tolls, while the suspicious one is ambivalent toward a rushed vaccination campaign with what are considered to be experimental vaccines, that is seen by some as the true goal of the “plandemic.” Without vaccination, the Covid-convinced future is projecting death at unprecedented scale, while the Covid-suspicious one is projecting lasting herd immunity acquired through natural body reactions. There are also alternative pasts. In the Covid-convinced history we are repeating the mistakes and suffering the fate of the Spanish Flu. In the suspicious counterpart, we should react like we did in the Hong-Kong Flu, but we are continuing instead a history of state subjugation to globalization and manipulation from global elites. In addition, there are diverging definitions of the moving present. Among the Covid-convinced, during 2020, the worst was yet to come, but, among the Covid-suspicious, we had rounded the corner and we had already vanquished the pandemic. The present in which thousands of people are dying daily from the pandemic co-existed with a present with no real Covid deaths, but just manipulation of evidence.

**FIGURE 7:**
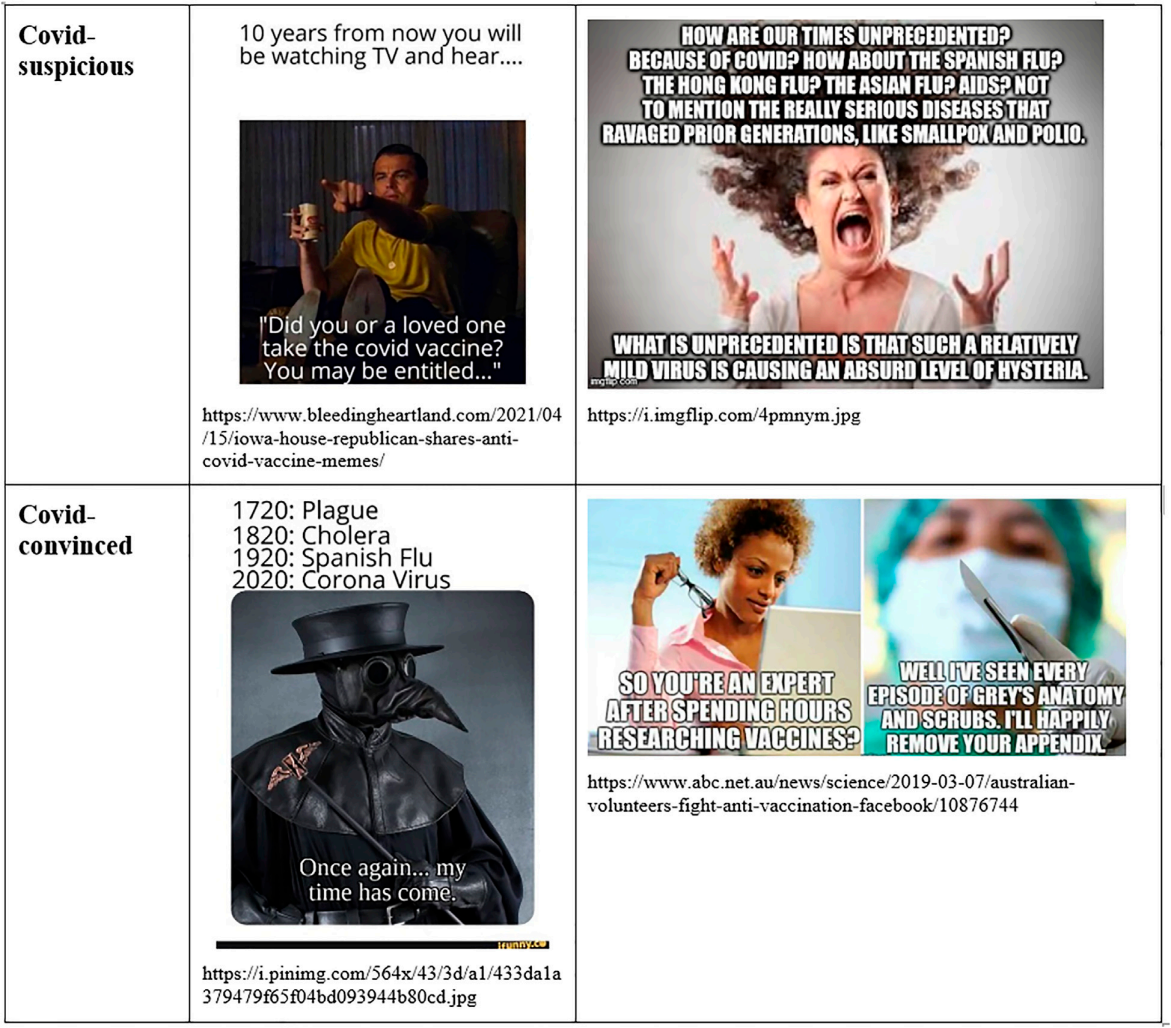
Time work in Covid-suspicious and Covid-convinced memes.

Time work is also visibly at play in public controversies on Covid therapies and vaccination (see Figure 7). The Hydroxychloroquine controversy pitted a medicine with a long history of use, very low costs, and connections to the even older life-saving remedy of quinine, against the recently developed, and quite expensive Remdesivir (Rughiniş et al., 2020). A similar controversy evolved around Ivermectin, another low-cost, high-promise drug with a prestigious history (Aeschlimann, 2021). The pursuit of vaccines has involved considerable debate about how to accelerate the process without compromising safety, efficacy, and public trust (Poland, 2020). The “Operation Warp Speed” policy and multiple declarations and anticipations of President Donald Trump highlighted tempo. The Russian authorities approved the public rollout of the Gamaleya vaccine before the completion of Phase III trials, while mainstream experts decried the rush (Callaway, 2020). Meanwhile, nine pharmaceutical companies racing to complete a vaccine struggled to avoid the suspicions associated with rapid production by signing a pledge for safety (Signatories of the Petition, 2020). The unprecedented speed of development of Covid-19 vaccines has remained a topic of divergent interpretations, being either a mark of danger for barely tested, experimental interventions, or a mark of the spectacular success of present-day science.

## Emotion Work

As regards *emotion work*, fear plays an important role on both sides of these controversies. While vaccine-distrusting views fear vaccine adverse effects and, as a rule, push away fears against preventable diseases, the vaccine-trusting views mandate fear of disease and dissuade fear of vaccine risks. Vaccine-distrusting views are often accused of fear-mongering. Examples can be found in the BBC’s report on antivaxx memes (Goodman and Carmichael, 2020). In a related vein, Lawrence highlights the special role of “fear of the irreparable” as an argument for quality, rather than quantity, used across the vaccination controversy to considerable rhetorical effect (Lawrence, 2016). At the same time, pro-vaccination discourses also appeal to fear against the dread of preventable diseases, and with the hope of eradicating them through vaccination. A study on vaccination memes found that pro-vaccine memes were appealing to sarcasm more often, a mark of boundary work, while anti-vaccination memes were using more fear (Harvey et al., 2019).

**FIGURE 8:**
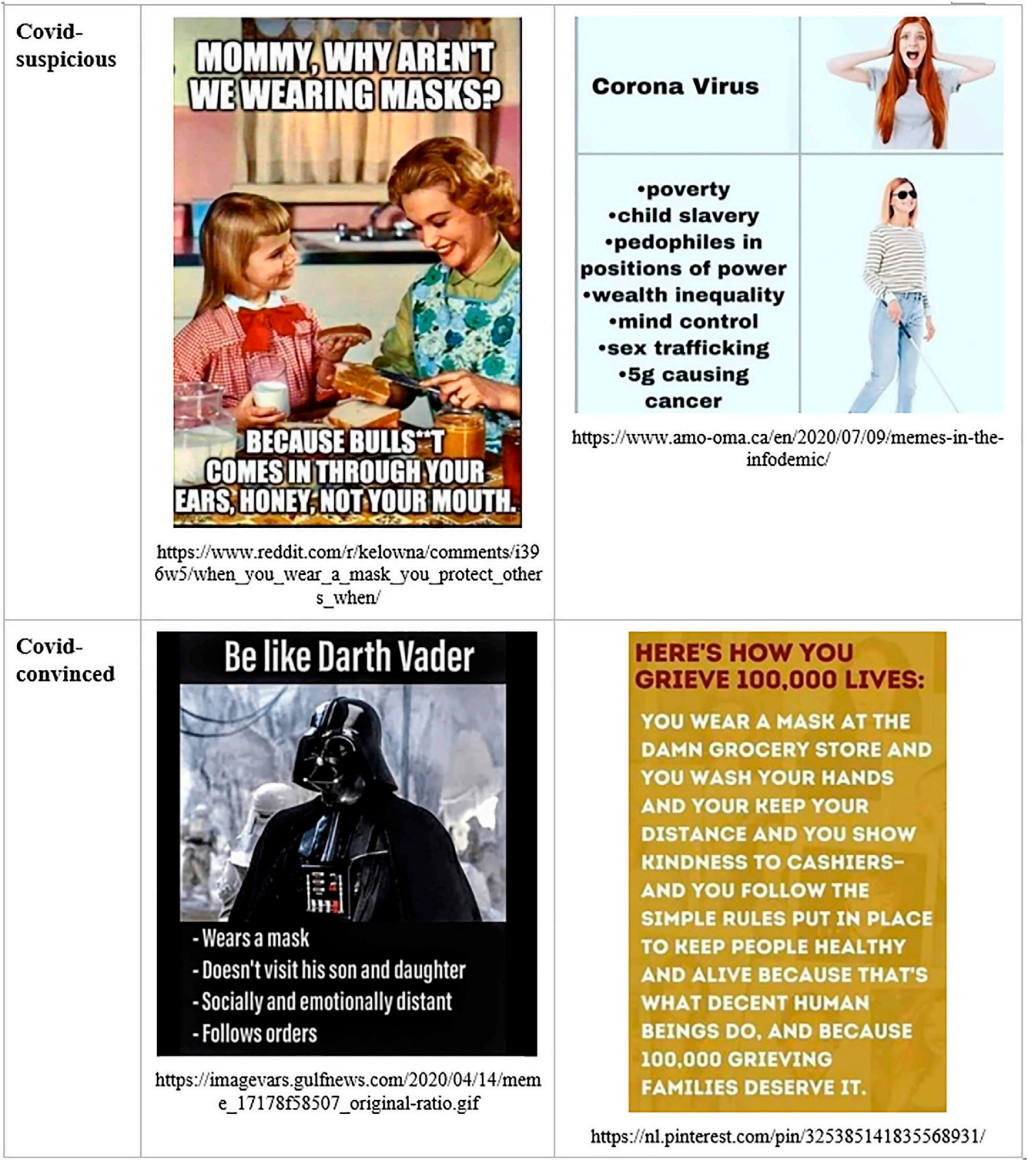
Emotion work in covid-skeptical and covid-anxious memes.

The *emotion work* differs markedly in the corona-convinced and corona-suspicious definitions of the situation (see Figure 8). In the corona-convinced view, everybody is encouraged to fear the novel coronavirus, despite great differences in the mortality rate. Taking infection risks is blamed because it is selfish, stupid, and irresponsible by harming others. We should trust experts, and we should trust masks. The inconvenience of masks is not worth getting angry about. Mask requirements are not infringements on individual freedom but justified limitations, in order to preserve others’ right to life. Alternatively, in the corona-suspicious view, the novel coronavirus is not to be feared. Everybody should face it and handle the risk individually and reasonably, as we do with the seasonal flu. Accepting the Covid risks to save the economy and people’s livelihoods is heroic. We should not let the cure be worse than the disease. We should feel righteous anger at the malevolence and incompetence of experts, mainstream media, and politicians. We should despise masks. There is also symmetrical gendering of masks across the two definitions of the situation. The corona-suspicious public push a gendered portrayal of mask-wearers as weak, submissive, and fearful, especially resonating with men’s concerns, while the corona-convinced public denounce the “toxic masculinity” of mask refusal, as illustrated by Capraro and Barcelo (2020).

Emotion work is important for establishing alliances across different definitions of the situation. Similar feeling rules may unite communities with different medical ontologies. For example, scientifically minded libertarians and conspiracy-minded corona-skeptics share a disapproval of state intervention and a policy of lockdowns and restrictions, cultivating similar emotional inclinations that make them likely political allies.

## Boundary Work

For each side in a science-related controversy, the others are portrayed as being duped, naïve, led by emotions rather than reason, and misunderstanding authentic science. Each side blames the other for manipulative uses of emotions and cherry-picked evidence, in symmetrical forms of *boundary maintenance work*. For example, there is stigmatization of the outgroup for both vaccine-confident parents (Rozbroj et al., 2019; Toth, 2020) and vaccine-skeptical ones (Toth, 2019).

The corona-convinced public engages in *boundary work* by positing Covid-suspicious people (dubbed *Covidiots*) as misinformed and held captive by a defensive, rationalizing worldview, created by manipulative media and elites. In this view, scientific references of Covid-suspicious arguments are either fake, or come from a few scientists, most of them on the fringe of the profession. Not wearing a mask is seen as stupid, selfish, and offensive. Symmetrically, for corona-suspicious actors, the Covid-convinced people (dubbed *sheeple*) are posited as misinformed and held captive by a panicking, disenfranchising worldview created by manipulative mainstream media and elites. The scientific references of Covid-convinced arguments come from cherry-picked scientists, and from the mainstream science, which is hijacked by commercial and political interests. Wearing a mask is seen as stupid, while enforcing it on others is seen as both stupid and offensive. This symmetrical boundary work can be observed in the twin versions of the “Karen” mocked in corona-suspicious and corona-convinced memes, respectively (Bhasin et al., 2020).

**FIGURE 9:**
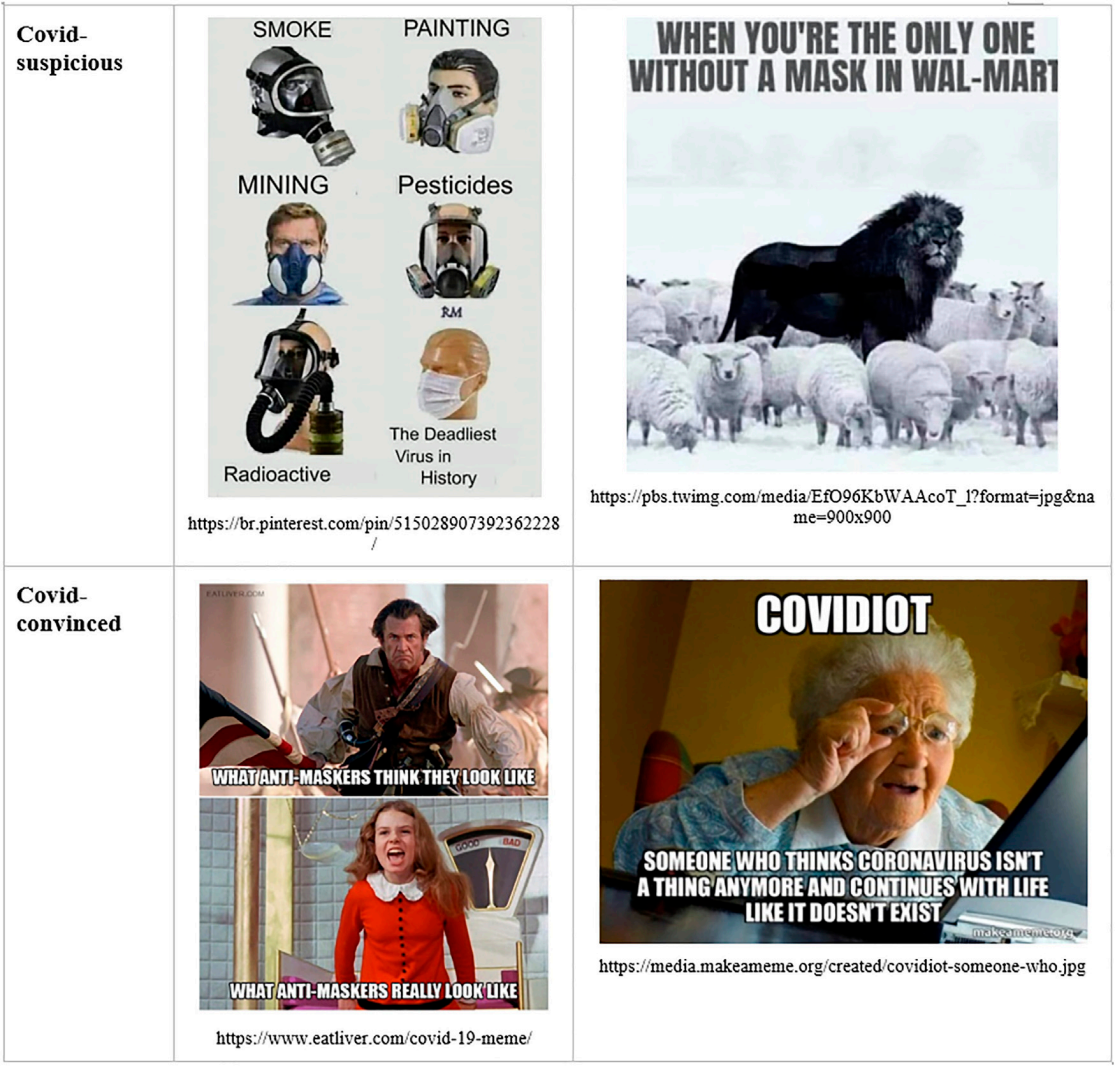
Boundary maintenance in Covid-skeptical and Covid-anxious memes.

**FIGURE 10:**
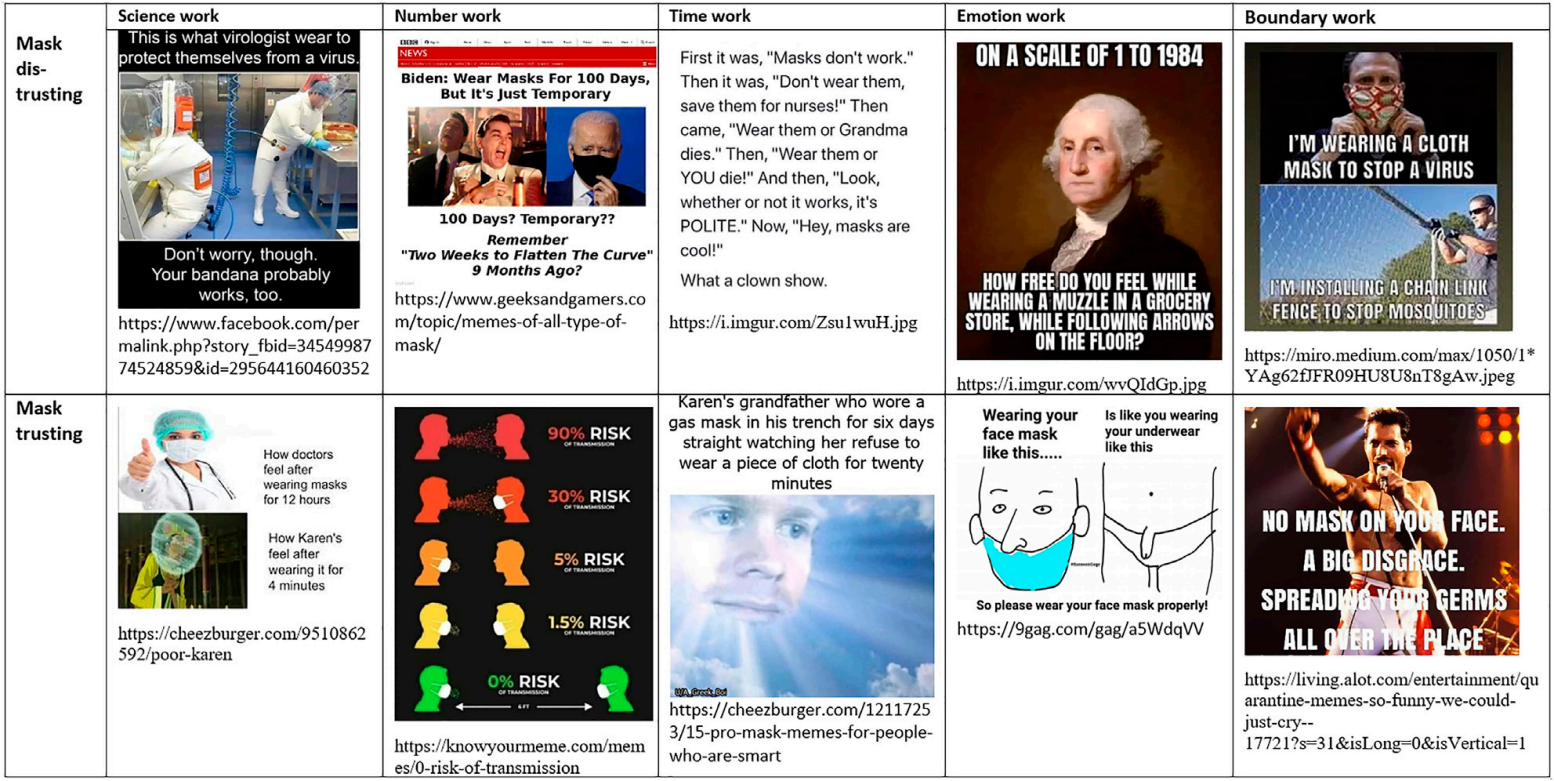
Social construction of masks as hinge objects through science work, number work, time work, emotion work, and boundary work.

We can also notice, in Figures 9 and 10, how the sanitary mask has become, again, a hinge object during the Covid-19 pandemic, after a similar development during the Spanish Flu (Hauser, 2020). Masks are seen as either tools of protection or vehicles of toxicity and political manipulation. Science work, number work, emotion work, time work, and boundary work converge to sustain the respective interpretations.

## Discussion

In this paper, we analyze five concepts and we synthesize a conceptual template for the symmetrical study of the social bifurcation of reality, focusing on the social construction of knowledge in science-distrusting and science-trusting worldviews. We illustrate the template in relation to the vaccination controversies and with polarized views of the Covid-19 pandemic. The template builds on five sensitizing concepts that capture agency in the social construction of knowledge: science work or staging science (Degele, 2005), number work or quantification (Espeland and Stevens, 2009), time work (Flaherty, 2003; Flaherty, 2011), emotion work (Hochschild, 1979), and boundary work or maintenance (Lamont and Molnár, 2002).

This template is useful in allowing observers, including scholars and journalists, an alternative to the deficit model (Gross, 1994; Miller, 2001) when examining public engagement with science. Rather than focusing on portraying the public as “faulty scientists” (Locke, 2002), the template encourages the observation of rhetorical innovations and alliances that establish or maintain specific worldviews across multiple communication media.

Moreover, a symmetrical analysis of science-distrusting and science-trusting worldviews leads to significant challenges in accounting for the emergence of science-distrusting views as obvious derivatives of postmodernism. At both ends of the mainstream science-confidence spectrum, actors search for objective, factual truths and criticize their opponents for espousing ideological “narratives,” thus departing from the relativistic epistemologies specific to postmodern thinking.

This conceptual template is also useful to explicate the emergence of *hinge objects,* or controversial sociotechnical objects—such as guns, cigarettes, vaccines, or masks—and their role in the social construction of knowledge. Hinge objects articulate bifurcated realities in the making, uniting them through shared elements and differentiating them through divergent interpretations. We aim to study them in future research, in counter-distinction to the *boundary objects* (Star and Griesemer, 1989; Star, 2010), which mediate collaboration across communities of practice.

As Figure 10 illustrates, all five processes discussed in this article contribute to the social bifurcation of reality and to the emergence of hinge objects, with many intersections. Science work and number work are useful to establish the *ontology* of an ideal typical discourse, answering questions such as the following: What are the relevant forces that shape the world? What are the most important causes and their effects? Conversely, time work, emotion work, and boundary work are useful to delineate the *narrative* that animates this ontology, through character construction and stories about past, present, and possible futures. Yet there is significant overlap. The natural ontology establishes what is *risky* and, thus, legitimates feeling rules: What should we fear? What should we pursue? The political narrative establishes who is *dangerous* and, thus, also formulates feeling rules: Whom should we fear? Whom should we trust? Social actors use time work and emotion work to sketch biographies of heroes and antagonists, highlighting their deeds and misdeeds, including the experts who may be deemed trustworthy and knowledgeable, or quite the opposite (See Figure 2). By creating and maintaining boundaries between the honest and the corrupt experts, or the competent and the incompetent ones, social actors select, in turn, the sources of scientific evidence and numbers that consolidate their ontology (see Figures 4, 6). Time work, emotion work, and boundary work are also useful to make sense of our experiences, and thus to feel the appropriate emotions and make allies with the right people. Should we feel relieved, grateful, amused, worried, disdainful, outraged, or something else altogether, when somebody wears a mask, carries a gun, shows proof of vaccination, or lights a cigarette in our presence? Should we express our feelings, or keep them to ourselves? Answers depend on what forces we oppose, what experiences we have, their historical significance, the future they portend, and our individual and collective powers.

Science is instantiated differently across divergent discourses, as facts and findings are brought forward by various actors claiming expertise, while challenging alternative accounts. Key objects acquire diverging interpretations and forms, becoming hinge objects that articulate worldviews and enable their further polarization. Pursuing truth and avoiding manipulation involves science work and number work, but also historical assessment through time work, making difficult choices relying on emotion work, and choosing sides through boundary work. Future research could clarify the ways in which specific social actors, such as lay individuals, professionals, or organizations, selectively deploy these processes, in combination, to shape their worldviews and to navigate controversial topics and complicated choices.

## References

[B1] Aeschlimann J. R. (2021). Ivermectin Is a Nobel Prize-Winning Wonder Drug – but Not for COVID-19. The Conversation. Available at: https://theconversation.com/ivermectin-is-a-nobel-prize-winning-wonder-drug-but-not-for-covid-19-168449 (Accessed January 5, 2021).

[B2] Alwan N. A. Burgess R. A. Ashworth S. Beale R. Bhadelia N. Bogaert D. (2020). Scientific Consensus on the COVID-19 Pandemic: We Need to Act Now. The Lancet 396 (10260), e71. 10.1016/S0140-6736(20)32153-X PMC7557300 33069277

[B3] Attwell K. Ward P. R. Meyer S. B. Rokkas P. J. Leask J. (2018). "Do-it-yourself": Vaccine Rejection and Complementary and Alternative Medicine (CAM). Soc. Sci. Med. 196, 106–114. 10.1016/j.socscimed.2017.11.022 29175699

[B4] Bakker J. I. H. (2016). “Definition of the Situation,” in The Blackwell Encyclopedia of Sociology (Oxford, UK: John Wiley & Sons), 1–2. 10.1002/9781405165518.wbeosd012.pub2

[B5] Bearman P. (2010). Just-so Stories: Vaccines, Autism, and the Single-Bullet Disorder. Soc. Psychol. Q. 73 (2), 112–115. 10.1177/0190272510371672 PMC2922865 20721312

[B6] Becker H. S. (1963). Outsiders. New York: Free Press.

[B7] Berger P. L. Thomas L. (1966). The Social Construction of Reality: A Treatise in the Sociology of Knowledge Garden City, NY: Anchor Books.

[B8] Bhasin T. Butcher C. Gordon E. Hallward M. LeFebvre R. (2020). Does Karen Wear a Mask? the Gendering of COVID-19 Masking Rhetoric. Ijssp 40, 929–937. 10.1108/IJSSP-07-2020-0293

[B9] Billig M. (2021). Rhetorical Uses of Precise Numbers and Semi-magical Round Numbers in Political Discourse about COVID-19: Examples from the Government of the United Kingdom. Discourse Soc. 32, 542–558. 10.1177/09579265211013115

[B10] Bloor D. (1976). Knowledge and Social Imagery. London: Routledge & Kegan Paul.

[B11] Blume S. (2006). Anti-Vaccination Movements and Their Interpretations. Soc. Sci. Med. 62 (3), 628–642. 10.1016/j.socscimed.2005.06.020 16039769

[B12] Bocquier A. Fressard L. Cortaredona S. Zaytseva A. Ward J. Gautier A. (2018). Social Differentiation of Vaccine Hesitancy Among French Parents and the Mediating Role of Trust and Commitment to Health: A Nationwide Cross-Sectional Study. Vaccine 36 (50), 7666–7673. 10.1016/j.vaccine.2018.10.085 30391054

[B13] Brekhus W. (1998). A Sociology of the Unmarked: Redirecting Our Focus. Sociological Theor. 16 (1), 34–51. 10.1111/0735-2751.00041

[B14] Bricker B. Justice J. (2019). The Postmodern Medical Paradigm: A Case Study of Anti-MMR Vaccine Arguments. West. J. Commun. 83 (2), 172–189. 10.1080/10570314.2018.1510136

[B15] Brown R. H. (1990). Rhetoric, Textuality, and the Postmodern Turn in Sociological Theory. Sociological Theor. 8 (2), 188. 10.2307/202204

[B16] Browne M. Thomson P. Rockloff M. J. Pennycook G. (2015). Going against the Herd: Psychological and Cultural Factors Underlying the ‘Vaccination Confidence Gap'. PLOS ONE 10 (9), e0132562. 10.1371/journal.pone.0132562 26325522 PMC4556675

[B17] Bulkeley H. (2000). Common Knowledge? Public Understanding of Climate Change in Newcastle, Australia. Public Underst Sci. 9 (3), 313–333. 10.1177/096366250000900301

[B18] Callaway E. (2020). Russia's Fast-Track Coronavirus Vaccine Draws Outrage over Safety. Nature 584, 334–335. 10.1038/d41586-020-02386-2 32782400

[B19] Capraro V. Barcelo H. (2020). “The Effect of Messaging and Gender on Intentions to Wear a Face Covering to Slow Down COVID-19 Transmission”. arXiv preprint. arXiv:2005.05467. Available at: https://arxiv.org/ftp/arxiv/papers/2005/2005.05467.pdf (Accessed October 1, 2021). 10.1002/acp.3793 PMC8013666 33821089

[B20] Carrion M. L. (2018). “You Need to Do Your Research”: Vaccines, Contestable Science, and Maternal Epistemology. Public Underst. Sci. 27 (3), 310–324. 10.1177/0963662517728024 28841813

[B22] CDC - Centers for Disease Control and Prevention (2021a). Selected Adverse Events Reported after COVID-19 Vaccination. Available at: https://www.cdc.gov/coronavirus/2019-ncov/vaccines/safety/adverse-events.html (Accessed January 5, 2022).

[B21] CDC - Centers for Disease Control and Prevention (2021b). Excess Deaths Associated with COVID-19. Available at: https://www.cdc.gov/nchs/nvss/vsrr/covid19/excess_deaths.htm (Accessed January 5, 2022).

[B23] Children’s Health Defense Team (2021). “One-Third of Deaths Reported to CDC after COVID Vaccines Occurred within 48 hours of Vaccination”. The Defender. Available at: https://childrenshealthdefense.org/defender/latest-data-cdc-vaers/ (Accessed March 14, 2021).

[B24] Ciocănel A. Rughiniş C. Flaherty M. G. (2020). Argumentative Time Work for Legitimizing Homeopathy: Temporal Reasons for the Acceptance of an Alternative Medical Practice. Time Soc. 30, 100–125. 10.1177/0961463X20962663

[B25] Ciocănel A. (2016). “A Remedy that Suits Me”: Classification of People and Individualization in Homeopathic Prescribing. J. Comp. Res. Anthropol. Sociol. 702, 113–124.

[B26] Coleman M. C. (2017). Rhetorical Logic Bombs and Fragmented Online Publics of Vaccine Science. J. Contemp. Rhetoric 7 (4), 203–216.

[B27] Collins H. Evans R. (2008). Rethinking Expertise. Chicago: University of Chicago Press.

[B28] Dalsgård A. L. Frederiksen M. Hojlund S. Meinert L. (Editors) (2014). Ethnographies of Youth and Temporality: Time Objectified (Philadelphia: Temple University Press).

[B29] Damasio A. R. (2005). Descartes’ Error: Emotion, Reason, and the Human Brain. London: Penguin Books.

[B30] Davies S. R. (2019). Science Communication as Emotion Work: Negotiating Curiosity and Wonder at a Science Festival. Sci. as Cult. 28 (4), 538–561. 10.1080/09505431.2019.1597035

[B31] Degele N. (2005). On the Margins of Everything: Doing, Performing and Staging Science in Homeopathy. Sci. Tech. Hum. Values 30 (1), 113–136. 10.1177/0162243904270711

[B32] Doty C. (2015). “Social Epistemology and Cognitive Authority in Online Comments about Vaccine Safety,” in iConference 2015 Proceedings, Newport Beach, CA, USA,, March 24–27, 2015 (iSchools).

[B33] Durnová A. (2018). Understanding Emotions in Policy Studies through Foucault and Deleuze. PaG 6 (4), 95–102. 10.17645/pag.v6i4.1528

[B34] Durnová A. (2019). Unpacking Emotional Contexts of Post-Truth. Crit. Pol. Stud. 13 (4), 447–450. 10.1080/19460171.2019.1670222 PMC7194248 32406401

[B35] Emirbayer M. Mische A. (1998). What Is Agency? Am. J. Sociol. 103 (4), 962–1023. 10.1086/231294

[B36] Emirbayer M. Sheller M. (1999). Publics in History. Theor. Soc. 28 (1), 145–197. 10.1023/a:1006921411329

[B37] Erikson K. (2017). The Sociologist’s Eye. London: Yale University Press.

[B38] Espeland W. N. Stevens M. L. (2009). A Sociology of Quantification. Eur. J. Sociol. 49 (03), 401. 10.1017/S0003975609000150

[B39] Eyal G. (2013). For a Sociology of Expertise: The Social Origins of the Autism Epidemic. Am. J. Sociol. 118 (4), 863–907. 10.1086/668448

[B40] Fischer F. (2019). Knowledge Politics and Post-Truth in Climate Denial: On the Social Construction of Alternative Facts. Crit. Pol. Stud. 13 (2), 133–152. 10.1080/19460171.2019.1602067

[B41] Fischer F. (2020). Post-truth Politics and Climate Denial: Further Reflections. Crit. Pol. Stud. 14 (1), 124–130. 10.1080/19460171.2020.1734846

[B42] Flaherty M. G. Meinert L. Dalsgård A. L. (2020). Time Work: Studies of Temporal Agency. New York: Berghahn Books.

[B43] Flaherty M. G. (2011). The Textures of Time Agency and Temporal Experience. Philadelphia: Temple University Press.

[B44] Flaherty M. G. Denzin N. K. Manning P. K. Snow D. A. (2002). Review Symposium. J. Contemp. Ethnography 31 (4), 478–516. 10.1177/0891241602031004004

[B45] Flaherty M. G. Rughiniş C. (2021). Online Memes and COVID-19. Contexts 20, 40–45. Available at: https://journals.sagepub.com/doi/full/10.1177/15365042211035338 (Accessed August 25, 2021). 10.1177/15365042211035338

[B46] Flaherty M. G. (2003). Time Work: Customizing Temporal Experience. Soc. Psychol. Q. 66 (1), 17–33. 10.2307/3090138

[B47] Fligstein N. (2001). Social Skill and the Theory of Fields. Sociological Theor. 19 (2), 105–125. 10.1111/0735-2751.00132

[B48] Gasparini R. Panatto D. Lai P. L. Amicizia D. (2015). The “urban Myth” of the Association between Neurological Disorders and Vaccinations. J. Prev. Med. Hyg. 56 (1), E1–E8. 26789825 PMC4718347

[B49] Gerber J. S. Offit P. A. (2009). Vaccines and Autism: A Tale of Shifting Hypotheses. Clin. Infect. Dis. 48 (4), 456–461. 10.1086/596476 19128068 PMC2908388

[B50] Goes L. J. (2013). “30 Scientific Studies Showing the Link between Vaccines and Autism.” Health Impact News. Available at: https://healthimpactnews.com/2013/30-scientific-studies-showing-the-link-between-vaccines-and-autism/ (Accessed March 14, 2021).

[B51] Goffman E. (1986). Frame Analysis. An Essay on the Organization of Experience. Lebanon: University Press of New England.

[B52] Goldberg R. F. Vandenberg L. N. (2019). Distract, Delay, Disrupt: Examples of Manufactured Doubt from Five Industries. Rev. Environ. Health 34 (4), 349–363. 10.1515/reveh-2019-0004 31271562

[B53] Goldenberg M. J. (2016). Public Misunderstanding of Science? Reframing the Problem of Vaccine Hesitancy. Perspect. Sci. 24 (5), 552–581. 10.1162/POSC_a_00223

[B54] Goodman J. Carmichael F. (2020). “Covid-19: What’s the Harm of ‘Funny’ Anti-vaccine Memes?” BBC News. Available at: https://www.bbc.com/news/55101238 (Accessed December 3, 2020).

[B55] Gov.uk–The Government of the United Kingdom (2021). Coronavirus Vaccine - Weekly Summary of Yellow Card Reporting. Updated 24 December 2021. Available at: https://www.gov.uk/government/publications/coronavirus-covid-19-vaccine-adverse-reactions/coronavirus-vaccine-summary-of-yellow-card-reporting#analysis-of-data (Accessed January 5, 2022).

[B56] Gray J. A. (1999). Postmodern Medicine. Lancet 354 (9189), 1550–1553. 10.1016/s0140-6736(98)08482-7 10551517

[B57] Green A. (2017). “Going Viral in the Online Anti-vaccine Wars.” Wellcome Collection. Available at: https://wellcomecollection.org/articles/Whf_BSkAACsAgwil (Accessed December 11, 2020).

[B58] Gross A. G. (1994). The Roles of Rhetoric in the Public Understanding of Science. Public Underst. Sci. 3 (1), 3–23. 10.1088/0963-6625/3/1/001

[B59] Hadjipanayis A. van Esso D. del Torso S. Dornbusch H. J. Michailidou K. Minicuci N. (2020). Vaccine Confidence Among Parents: Large Scale Study in Eighteen European Countries. Vaccine 38 (6), 1505–1512. 10.1016/j.vaccine.2019.11.068 31848051

[B60] Hallett T. (2003). Emotional Feedback and Amplification in Social Interaction. Sociological Q. 44 (4), 705–726. 10.1111/j.1533-8525.2003.tb00532.x

[B61] Harvey A. M. Thompson S. Lac A. Coolidge F. L. (2019). Fear and Derision: A Quantitative Content Analysis of Provaccine and Antivaccine Internet Memes. Health Educ. Behav. 46 (6), 1012–1023. 10.1177/1090198119866886 31789076

[B62] Hauser C. (2020). The Mask Slackers of 1918. New York Times. Available at: https://www.nytimes.com/2020/08/03/us/mask-protests-1918.html (Accessed January 5, 2022).

[B63] Hausman B. L. (2019). Anti/Vax: Reframing the Vaccination Controversy. London: ILR Press.

[B64] Hirschman D. Reed I. A. (2014). Formation Stories and Causality in Sociology. Sociological Theor. 32 (4), 259–282. 10.1177/0735275114558632

[B65] Hobson-West P. (2003). Understanding Vaccination Resistance: Moving beyond Risk. Health Risk Soc. 5 (3), 273–283. 10.1080/13698570310001606978

[B66] Hobson-West P. Hons M. A. (2005). Understanding Resistance to Childhood Vaccination in the UK : Radicals , Reformists and the Discourses of Risk , Trust and Science. Doctoral thesis Nottingham, (UK): University of Nottingham.

[B67] Hochschild A. R. (1979). Emotion Work, Feeling Rules, and Social Structure. Am. J. Sociol. 85 (3), 551–575. 10.1086/227049

[B68] Hornsey M. J. Harris E. A. Fielding K. S. (2018). The Psychological Roots of Anti-vaccination Attitudes: A 24-Nation Investigation. Health Psychol. 37 (4), 307–315. 10.1037/hea0000586 29389158

[B69] James W. (1982). “Belief and Perception of Reality,” in Pragmatism, the Classic Writings. Editor Thayer H. S. (Indianapolis: Hackett Publishing Company).

[B70] Jamison A. M. Broniatowski D. A. Dredze M. Wood-Doughty Z. Khan D. Quinn S. C. (2020). Vaccine-Related Advertising in the Facebook Ad Archive. Vaccine 38 (3), 512–520. 10.1016/j.vaccine.2019.10.066 31732327 PMC6954281

[B71] Jasper J. M. (2014). “Feeling-Thinking: Emotions as Central to Culture,” in Conceptualizing Culture in Social Movement Research. UK: Palgrave Macmillan, 23–44. 10.1057/9781137385796_2

[B72] Johnson N. F. Velásquez N. Restrepo N. J. Leahy R. Gabriel N. El Oud S. (2020). The Online Competition between Pro- and Anti-vaccination Views. Nature 582, 230–233. 10.1038/s41586-020-2281-1 32499650

[B73] Jones J. S. (2021). Covid-19 Has Killed More Americans Than the Civil War. How Do We Remember Them? the Washington Post. Available at: https://www.washingtonpost.com/outlook/2021/12/02/covid-19-has-killed-more-americans-than-civil-war-how-do-we-memorialize-them/ (Accessed January 5, 2022).

[B74] Jurkane-Hobein I. (2015). When Less Is More: On Time Work in Long-Distance Relationships. Qual. Sociol. 38 (2), 185–203. 10.1007/s11133-015-9304-5

[B75] Kata A. (2010). A Postmodern Pandora's Box: Anti-vaccination Misinformation on the Internet. Vaccine 28 (7), 1709–1716. 10.1016/j.vaccine.2009.12.022 20045099

[B76] Kata A. (2012). Anti-vaccine Activists, Web 2.0, and the Postmodern Paradigm-Aan Overview of Tactics and Tropes Used Online by the Anti-vaccination Movement. Vaccine 30 (25), 3778–3789. 10.1016/j.vaccine.2011.11.112 22172504

[B77] Kennedy A. LaVail K. Nowak G. Basket M. Landry S. (2011). Confidence about Vaccines in the United States: Understanding Parents' Perceptions. Health Aff. (Millwood) 30 (6), 1151–1159. 10.1377/hlthaff.2011.0396 21653969

[B78] Kitta A. Goldberg D. S. (2017). The Significance of Folklore for Vaccine Policy: Discarding the Deficit Model. Crit. Public Health 27 (4), 506–514. 10.1080/09581596.2016.1235259

[B79] Krishna A. (2017). Motivation with Misinformation: Conceptualizing Lacuna Individuals and Publics as Knowledge-Deficient, Issue-Negative Activists. J. Public Relations Res. 29 (4), 176–193. 10.1080/1062726X.2017.1363047

[B80] Kulldorff M. Gupta S. Bhattacharya J. (2020). “The Great Barrington Declaration.” American Institute for Economic Research. Available at: https://gbdeclaration.org/ (Accessed November 1, 2020).

[B81] LaCour M. Davis T. (2020). Vaccine Skepticism Reflects Basic Cognitive Differences in Mortality-Related Event Frequency Estimation. Vaccine 38, 3790–3799. 10.1016/j.vaccine.2020.02.052 32169393

[B82] Lamont M. Molnár V. (2002). The Study of Boundaries in the Social Sciences. Annu. Rev. Sociol. 28 (1), 167–195. 10.1146/annurev.soc.28.110601.141107

[B83] Larson H. J. de Figueiredo A. Xiahong Z. Schulz W. S. Verger P. Johnston I. G. (2016). The State of Vaccine Confidence 2016: Global Insights through a 67-Country Survey. EBioMedicine 12, 295–301. 10.1016/j.ebiom.2016.08.042 27658738 PMC5078590

[B84] Lawrence H. Y. (2016). Fear of the Irreparable: Narratives in Vaccination Rhetoric. Narrative Inq. Bioeth. 6 (3), 205–209. 10.1353/nib.2016.0060

[B85] Locke S. (2002). The Public Understanding of Science-A Rhetorical Invention. Sci. Technol. Hum. Values 27 (1), 87–111. 10.1177/016224390202700104

[B86] Lois J. (2010). The Temporal Emotion Work of Motherhood. Gend. Soc. 24 (4), 421–446. 10.1177/0891243210377762

[B87] Massey D. S. (2002). A Brief History of Human Society: The Origin and Role of Emotion in Social Life: 2001 Presidential Address. Am. Sociological Rev. 67 (1), 1–29. 10.2307/3088931

[B88] Matei Ş. Preda M. (2019). When Social Knowledge Turns Mathematical - the Role of Formalisation in the Sociology of Time. Time Soc. 28 (1), 247–272. 10.1177/0961463X17752279

[B89] McCoy L. (2009). Time, Self and the Medication Day: a Closer Look at the Everyday Work of ‘adherence'. Sociol. Health Illness 31 (1), 128–146. 10.1111/j.1467-9566.2008.01120.x 19170973

[B90] Melley T. (2002). “Agency Panic and the Culture of Conspiracy,” in Conspiracy Nation: The Politics of Paranoia in Postwar America. Editor Knight P. (New York: New York University Press), 57–81.

[B91] Michael M. (2002). Comprehension, Apprehension, Prehension: Heterogeneity and the Public Understanding of Science. Sci. Technol. Hum. Values 27 (3), 357–378. 10.1177/016224390202700302

[B92] Michaels D. (2020). The Triumph of Doubt: Dark Money and the Science of Deception. Oxford: Oxford University Press.

[B93] Miller S. (2001). Public Understanding of Science at the Crossroads. Public Underst. Sci. 10 (1), 115–120. 10.3109/a036859

[B94] Moran M. B. Lucas M. Everhart K. Morgan A. Prickett E. (2016). What Makes Anti-vaccine Websites Persuasive? A Content Analysis of Techniques Used by Anti-vaccine Websites to Engender Anti-vaccine Sentiment. J. Commun. Healthc. 9 (3), 151–163. 10.1080/17538068.2016.1235531

[B95] Motta M. Stecula D. (2021). “Unverified Reports of Vaccine Side Effects in VAERS Aren’t the Smoking Guns Portrayed by Right-wing media Outlets – They Can Offer Insight into Vaccine Hesitancy”. The Conversation. Available at: https://theconversation.com/unverified-reports-of-vaccine-side-effects-in-vaers-arent-the-smoking-guns-portrayed-by-right-wing-media-outlets-they-can-offer-insight-into-vaccine-hesitancy-166401 (Accessed January 5, 2021).

[B96] Mukhtar S. (2020). Psychology and Politics of COVID-19 Misinfodemics: Why and How Do People Believe in Misinfodemics? Int. Sociol. 36, 111–123. 10.1177/0268580920948807

[B97] Mulimani P. S. (2017). Evidence-Based Practice and the Evidence Pyramid: A 21st Century Orthodontic Odyssey. Am. J. Orthod. Dentofacial Orthop. 152 (1), 1–8. 10.1016/j.ajodo.2017.03.020 28651753

[B98] Napolitano F. D'Alessandro A. Angelillo I. F. (2018). Investigating Italian Parents' Vaccine Hesitancy: A Cross-Sectional Survey. Hum. Vaccin. Immunother. 14 (7), 1558–1565. 10.1080/21645515.2018.1463943 29641945 PMC6067864

[B99] Nazar S. Pieters T. (2021). Plandemic Revisited: A Product of Planned Disinformation Amplifying the COVID-19 “Infodemic”. Front. Public Health 9, 649930. 10.3389/fpubh.2021.649930 34336759 PMC8318131

[B100] Numerato D. Vochocová L. Štětka V. Macková A. (2019). The Vaccination Debate in the “post‐truth” Era: Social media as Sites of Multi‐layered Reflexivity. Sociol. Health Illn. 41 (S1), 82–97. 10.1111/1467-9566.12873 31599993

[B101] Orekes N. Conway E. (2010). Merchants of Doubt. New York: Bloomsbury Press.

[B102] Pachucki M. A. Pendergrass S. Lamont. M. (2007). Boundary Processes: Recent Theoretical Developments and New Contributions. Poetics 35 (6), 331–351. 10.1016/j.poetic.2007.10.001

[B103] Plutzer E. Hannah A. L. (2018). Teaching Climate Change in Middle Schools and High Schools: Investigating STEM Education's Deficit Model. Climatic Change 149 (3–4), 305–317. 10.1007/s10584-018-2253-8

[B104] Poland C. M. Poland G. A. (2011). Vaccine Education Spectrum Disorder: The Importance of Incorporating Psychological and Cognitive Models into Vaccine Education. Vaccine 29 (37), 6145–6148. 10.1016/j.vaccine.2011.07.131 21840462

[B105] Poland G. A. (2020). Tortoises, Hares, and Vaccines: A Cautionary Note for SARS-CoV-2 Vaccine Development. Vaccine 38 (27), 4219–4220. 10.1016/j.vaccine.2020.04.073 32387011 PMC7252125

[B106] Poltorak M. Leach M. Fairhead J. Cassell J. (2005). ‘MMR Talk' and Vaccination Choices: an Ethnographic Study in Brighton. Soc. Sci. Med. 61 (3), 709–719. 10.1016/j.socscimed.2004.12.014 15899328

[B107] Prior L. (2003). Belief, Knowledge and Expertise: The Emergence of the Lay Expert in Medical Sociology. Sociol. Health Illness 25 (3), 41–57. 10.1111/1467-9566.00339 14498929

[B108] Ramírez-i-Ollé M. (2018). Trust, Scepticism, and Social Order: A Contribution from the Sociology of Scientific Knowledge. Sociol. Compass 13 (2), e12653. 10.1111/soc4.12653

[B109] Reich J. A. (2014). Neoliberal Mothering and Vaccine Refusal. Gend. Soc. 28 (5), 679–704. 10.1177/0891243214532711

[B110] Rendle K. A. Leskinen E. A. (2017). Timing Is Everything: Exploring Parental Decisions to Delay HPV Vaccination. Qual. Health Res. 27 (9), 1380–1390. 10.1177/1049732316664499 27557924

[B111] Ritchie H. Ortiz-Espina D. B. Mathieu E. Joe H. Macdonald B. Giattino C. (2020). “Mortality Risk of COVID-19.” Our World in Data. Available at: https://ourworldindata.org/mortality-risk-covid (Accessed December 13, 2020).

[B112] Robison S. G. Groom H. Young C. (2012). Frequency of Alternative Immunization Schedule Use in a Metropolitan Area. Pediatrics 130 (1), 32–38. 10.1542/peds.2011-3154 22711719

[B113] Roozenbeek J. Schneider C. R. Dryhurst S. Kerr J. Freeman A. L. J. Recchia G. (2020). Susceptibility to Misinformation about COVID-19 Around the World. R. Soc. Open Sci. 7 (10), 201199. 10.1098/rsos.201199 33204475 PMC7657933

[B114] Rozbroj T. Lyons A. Lucke J. (2019). The Mad Leading the Blind: Perceptions of the Vaccine-Refusal Movement Among Australians Who Support Vaccination. Vaccine 37 (40), 5986–5993. 10.1016/j.vaccine.2019.08.023 31451326

[B115] Rughiniş C. Dima L. Vasile S. (2020). Hydroxychloroquine and COVID-19: Lack of Efficacy and the Social Construction of Plausibility. Am. J. Ther. 27 (6), e573. 10.1097/MJT.0000000000001294 33136577

[B116] Sanders C. Burnett K. (2019). The Neoliberal Roots of Modern Vaccine Hesitancy. J. Health Soc. Sci. 4 (2), 149–156.

[B117] Scott J. B. (2016). Boundary Work and the Construction of Scientific Authority in the Vaccines-Autism Controversy. J. Tech. Writing Commun. 46 (1), 59–82. 10.1177/0047281615600638

[B118] Signatories of the Petition (2020). “Biopharma Leaders Unite to Stand with Science. Nine CEOs Sign Historic Pledge to Continue to Make the Safety and Well-Being of Vaccinated Individuals the Top Priority in Development of the First COVID-19 Vaccines.” Businesswire. Available at: https://www.businesswire.com/news/home/20200908005498/en/ (Accessed October 10, 2020).

[B119] Simis M. J. Madden H. Cacciatore M. A. Yeo S. K. (2016). The Lure of Rationality: Why Does the Deficit Model Persist in Science Communication? Public Underst. Sci. 25 (4), 400–414. 10.1177/0963662516629749 27117768

[B120] Simmel G. (1909). The Problem of Sociology. Am. J. Sociol. 15 (3), 289–320. 10.1086/211783

[B121] Slater M. H. Huxster J. K. Bresticker J. E. LoPiccolo V. (2020). Denialism as Applied Skepticism: Philosophical and Empirical Considerations. Erkenn 85 (4), 871–890. 10.1007/s10670-018-0054-0

[B122] Smith D. E. (1996). Telling the Truth after Postmodernism1. Symbolic Interaction 19 (3), 171–202. 10.1525/si.1996.19.3.171

[B123] Smith E. (2021). How many People Have Died from the Vaccine in the U.S. Covid-101. Available at: https://covid-101.org/science/how-many-people-have-died-from-the-vaccine-in-the-u-s/ (Accessed January 5, 2022).

[B124] Star S. L. Griesemer J. R. (1989). Institutional Ecology, ‘Translations’ and Boundary Objects: Amateurs and Professionals in Berkeley's Museum of Vertebrate Zoology, 1907-39. Soc. Stud. Sci. 19 (3), 387–420. 10.1177/030631289019003001

[B125] Star S. L. (2010). This Is Not a Boundary Object: Reflections on the Origin of a Concept. Sci. Technol. Hum. Values 35 (5), 601–617. 10.1177/0162243910377624

[B126] Steele J. M. (2005). Darrell Huff and Fifty Years of ‘How to Lie with Statistics. Stat. Sci. 20 (3), 205–209. 10.1214/088342305000000205

[B127] StopMandatoryVaccination.com (2020). Vaccine Injury & Death Stories. Available at: https://www.stopmandatoryvaccination.com/vaccine-dangers/vaccine-injury-stories/ (Accessed December 12, 2020).

[B128] Stoutenborough J. W. Vedlitz A. (2014). The Effect of Perceived and Assessed Knowledge of Climate Change on Public Policy Concerns: An Empirical Comparison. Environ. Sci. Pol. 37, 23–33. 10.1016/j.envsci.2013.08.002

[B129] Suldovsky B. (2017). The Information Deficit Model and Climate Change Communication. Oxford Res. Ency. Climate Sci. 10.1093/ACREFORE/9780190228620.013.301

[B130] Thoits P. A. (2004). “Emotion Norms, Emotion Work, and Social Order,” in Feelings and Emotions: The Amsterdam Symposium. Editors Manstead A. S. R. Frijda N. Fischer A. (Cambridge, UK: Cambridge University Press), 359–378. 10.1017/cbo9780511806582.021

[B131] Thomas W. I. Thomas D. S. (1928). The Child in America. Behavior Problems and Programs. New York: Alfred A. Knopf.

[B132] Tomljenovic H. Bubic A. Erceg N. (2020). It Just Doesn't Feel Right - the Relevance of Emotions and Intuition for Parental Vaccine Conspiracy Beliefs and Vaccination Uptake. Psychol. Health 35 (5), 538–554. 10.1080/08870446.2019.1673894 31588791

[B133] Torcello L. (2016). The Ethics of Belief, Cognition, and Climate Change Pseudoskepticism: Implications for Public Discourse. Top. Cogn. Sci. 8 (1), 19–48. 10.1111/tops.12179 26799170

[B134] Toth C. (2019). The Rational, Loving and Responsible Parent. A Discursive Construction of the Identities of the Parents that Decided Not to Vaccinate Their Children. J. Comp. Res. Anthropol. Sociol. 10 (2), 1–14.

[B135] Toth C. (2020). To Vaccinate or Not to Vaccinate My Child?’ what Is at Stake in Vaccination Repertoires?” On_education. J. Res. Debate 3 (8), 1–6. 10.17899/on_ed.2020.8.8

[B137] Vulpe S.-N. (2020). Understanding Vaccine Hesitancy as Extended Attitudes. Eur. Rev. Appl. Sociol. 1320, 43–57. 10.1515/eras-2020-0005

[B136] Vulpe S.-N. (2021). Belief and Skepticism. Religious Justifications for Vaccine Reluctance and Climate Skepticism. Soc. Rom. 192, 69–88. 10.33788/sr.19.2.3

[B138] Wang Y. McKee M. Torbica A. Stuckler D. (2019). Systematic Literature Review on the Spread of Health-Related Misinformation on Social Media. Soc. Sci. Med. 240, 112552. 10.1016/j.socscimed.2019.112552 31561111 PMC7117034

[B139] Wardle C. Derakhshan H. (2017). Information Disorder: Toward an Interdisciplinary Framework for Research and Policy Making Strasbourg: Council of Europe.

[B140] Warraich H. (2020). “Covid-19 Is Creating a Wave of Heart Disease.”. The New York Times, August 17. Available at: https://www.nytimes.com/2020/08/17/opinion/covid-19-heartdisease.html (Accessed October 1, 2021).

[B141] Weber M. (1978). Economy and Society: An Outline of Interpretive Sociology. Editors Guenther R. Wittich C. (Berkeley: University of California Press).

[B142] Wynne B. (1996). “Misunderstood Misunderstandings: Social Identities and Public Uptake of Science,” in Misunderstanding Science? the Public Reconstruction of Science and Technology. Editors Irwin A. Wynne B. (Cambridge: Cambridge University Press), 19–46.

[B143] Yoo H. (2018). The Death of Expertise: The Campaign against Established Knowledge and Why it Matters. J. Korean Acad. Child. Adolesc. Psychiatry 29 (4), 185–186. 10.5765/jkacap.180021

[B144] Zerubavel E. (2018). Taken for Granted. Princeton, NJ: Princeton University Press.

[B145] Zingg A. Siegrist M. (2012). Measuring People's Knowledge about Vaccination: Developing a One-Dimensional Scale. Vaccine 30 (25), 3771–3777. 10.1016/j.vaccine.2012.03.014 22445808

